# Evaluation of cell penetrating peptide coated Mn:ZnS nanoparticles for paclitaxel delivery to cancer cells

**DOI:** 10.1038/s41598-018-20255-x

**Published:** 2018-01-30

**Authors:** N. Sanoj Rejinold, Yunho Han, Jisang Yoo, Hae Yong Seok, Ji Ho Park, Yeu-Chun Kim

**Affiliations:** 1 Department of Chemical and Biomolecular Engineering, Korea Advanced Institute of Science and Technology (KAIST), Daejeon, Republic of Korea; 2 Department of Brain and Bioengineering, Institute of Health Science and Technology, Korea Advanced Institute of Science and Technology (KAIST), Daejeon, Republic of Korea

## Abstract

This work aimed at formulating paclitaxel (PTX) loaded cell penetrating peptide (CPP) coated Mn doped ZnS nanoparticles (Mn:ZnS NPs) for improved anti-cancer efficacy *in vitro* and *in vivo*. The developed PTX loaded Mn:ZnS NPs with different CPPs (PEN, pVEC and R9) showed enhanced anti-cancer effect compared to bare PTX, which has been validated by MTT assay followed by apoptosis assay and DNA fragmentation analysis. The *in vivo* bio-distribution and anti-cancer efficacy was studied on breast cancer xenograft model showing maximum tumor localization and enhanced therapeutic efficacy with R9 coated Mn:ZnS NPs (R9:Mn:ZnS NPs) and was confirmed by H/E staining. Thus, R9:Mn:ZnS NPs could be an ideal theranostic nano-carrier for PTX with enhanced  the rapeutic efficacy toward cancer cells, where penetration and sustainability of therapeutics are essential.

## Introduction

Theranostic nanoparticles (NPs) are of great interest as they can impart both diagnosis and improve cancer therapy^1^. Theranostic NPs based on gold^2^ and iron oxide^3^ have been reported as biocompatible imaging agent in drug delivery. In recent years, many works have been employed to improve cancer-therapy by encapsulating drug molecules either in polymer carriers^4^ or inorganic NPs^5^.

Inorganic NPs such as Mn doped ZnS NPs (Mn:ZnS) have been widely used as imaging agent mainly due to their improved cytocompatibility^6^ and ROS generating capability^7^ compared to the undoped ZnS NPs. Inaddition, the Mn:ZnS could improve the *in vivo* clearance which is essential for drug delivery aplciations^7^. However, its low serum stability is still a major problem, limiting its potential in drug delivery. Recently aqueous synthesis of highly luminescent glutathione-modified Mn:ZnS NPs were developed by Kolmykov *et al*., 2014 without much experiments on stability which are crucial for bioapplications^8^. Similarly there have been various reports on preparative methos such as aqueous and non-aqueous routes for Mn:ZnS NPs^8^, however most of them used relatively toxic stabilizing agents. For example hemolytic chitosan was used to stabilize Mn:ZnS NPs by Bwatanglang., *et al*.^9^ and mercaptopropionic acid was used to synthesize Mn:ZnS NPs by Zhao *et al.*, 2015^10^.

Another study by Deng *et al*., 2011 reported a high-quality Mn:ZnS NPs with tunable dual-color and multiphoton emissions and could be useful for bioimaging. This study also lacks the stability of Mn:ZnS NPs^11^ This might be a possible reason for their limited use as drug delivery carrier except few reports such as folic acid targeted Mn:ZnS quantum dots for theranosis by Bwatanglang *et al*., 2016 which has no serum stability of Mn:ZnS NPs reported^9^. Zhao *et al*., 2017 reported that surface modification of Mn:ZnS NPs could improve the drug loading ability and optical properties^12^. Thus it is necessary to modify Mn:ZnS NPs with a proper passivating agents that could improve the serum stability and drug delivery efficiency specially at the *in vivo* level.

Cell penetrating peptides (CPPs) have been used for delivering genes and bio-macromolecules, as they can easily pass through the membrane in a non-endocytosis manner, making them attractive in drug delivery applications^13^. Thus coating Mn:ZnS NPs with CPPs might be ideal as they are cell penetrable whereas the cytocompatible ZnS based nano-crystals could be used only for *in vitro* bioimaging^14^. CPPs such as penetratin (PEN:originated from homeodomain of the *Drosophila* transcription factor Antennapedia^15^ with a sequence of RQIKIWFQNRRMKWKK) have been widely used for various biomedical applications^16^. Similarly, pVEC (Cadherin originated with amino acid sequence of LLIILRRRIRKQAHAHSK) and R9 (RRRRRRRRR) have potential for gene delivery applications^17^. Thus combining CPP and Mn:ZnS NPs could be beneficial for improved anti-cancer therapy as the Mn doping could enhance the ROS^7^ mediated apoptosis along with the Paclitaxel (PTX), a first line chemoagnet against many cancers such as breast (metastatic)^18^, ovarian^19^ and cervical cancers. Since UV fluoresced Mn:ZnS NPs has no live imaging capability, we further doped it with IR-780 dye to enhance the *in vivo* bio-imaging. Since the dye can be doped easily in the lattice space of Mn:ZnS NPs, the fluorescence of the dye can be retained for *in vivo* applications. Unlike the polymeric NPs, the Mn:ZnS NPs would render co-loading efficientlty as there might be minimum interaction beween PTX and IR-780 dye as the latter is inside the core space lattices while the former is attached on the CPP molecules on the shell.

**Figure 1:**
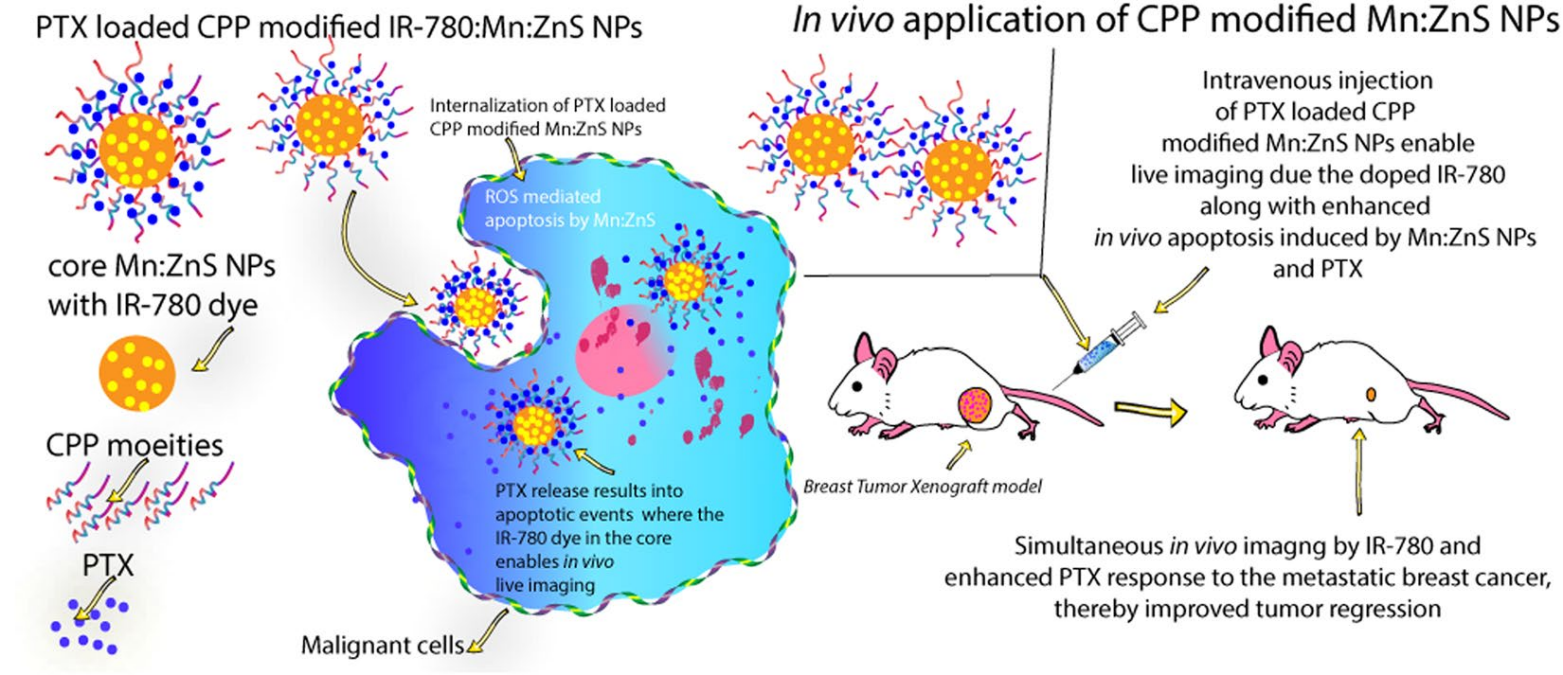
Research strategy for the CPP modified PTX loaded Mn:ZnS NPs for anti-cancer therapy. The Mn:ZnS NPs are doped with IR-780 dye in its core which can ensure enhanced *in vivo* live imaging, where the outer surface is coated with CPPs to improve the serum stability, and cell localization capability. The PTX molecules are attached with the coated CPPs and can be released in acidic environment where the Mn:ZnS NPs can induce ROS mediated apoptosis improving the PTX effect to the cancer cells.

Thus our current work focussed on modification of Mn:ZnS NPs for effective tumor cell penetration of partially soluble drugs like PTX for improving therapeutic outcome in breast cancer xenograft model along with *in vivo* imaging. The Mn:ZnS NPs were doped with IR-780 dye to have *in vivo* live imaging and coated with CPPs, maintaining their particle size in the optimum range for the PTX delivery. The basic research question we addressed in this study is: How can CPP modified IR-780:Mn:ZnS NPs act as an ideal theranostic agent for anticancer therapy *in vitro* and *in vivo*? We hypothesize that since Mn:ZnS NPs being cell illuminant and CPP, cell penetrable, they could improve the apoptosiss by PTX and ROS generated by the doped NPs, whereas the IR-780 in Mn:ZnS NPs core will enhance *in vivo* live imaging in cancer cells as strategized in Fig. 1.

## Results

### Synthesis, characterization, and PTX formulations with CPP coated Mn:ZnS NPs

**Figure 2:**
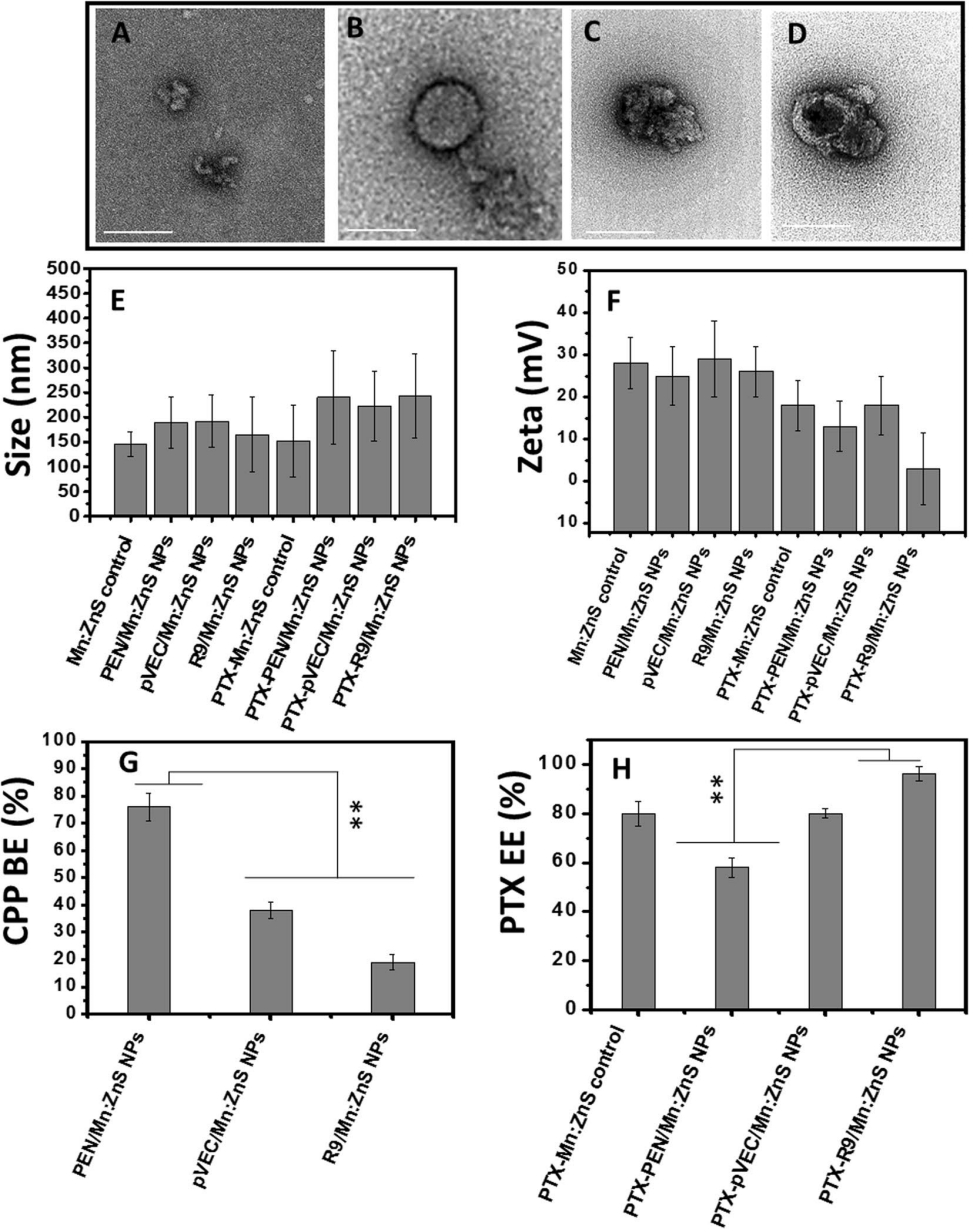
Surface morphology of (**A**) control Mn:ZnS NPs; (**B**) PEN modification on Mn:ZnS NPs; (**C**) pVEC modification on Mn:ZnS NPs; (**D**) R9 modification on Mn:ZnS NPs and respectively (scale bar-250 nm). (**E**) Size and (**F**) zeta potential assessment for CPP modified NPs with and without PTX loading; (**G**) CPP binding efficiency toward the Mn:ZnS NPs and (**H**) PTX encapsulation efficiency (n = 3, **represents the p < *0.01, BE = Binding efficiency and EE = Encapsulation efficiency)*.

Different CPP modified Mn:ZnS NPs have been prepared using a simple aqueous route in which CPP could act as a stabilizing and surface passivating agent. The CPP:Mn:ZnS NPs had almost spherical shapes, with size ranging from 100–150 nm, as visualized in the bio-TEM (Fig. 2B–D). Unlike the Mn:ZnS NPs (Fig. 2A), the CPP modified NPs showed distinct contrast in the bio-TEM, whereas the Mn:ZnS NPs appeared to have a darker core, while the CPP modification rendered a cloudy surface appearance.

In the case of the PEN modified NPs, the surface was completely covered compared to the other CPP molecules (Fig. 2B). The pVEC bound onto the Mn:ZnS NPs with an irregular covering(Fig. 2C). R9 showed entirely different attachment on the core, with maximum R9 residues attached on the side of Mn:ZnS NPs (Fig. 2D).

The DLS analysis showed slightly increased particle size, likely due to the swelling of CPP chains in aqueous solutions. The size varied from 130–230 nm for the control Mn:ZnS NPs, whereas the PEN: Mn:ZnS NPs showed 140–280 nm and pVEC modification had 140–240 nm (Fig. 2E). The R9:Mn:ZnS NPs showed 110–240 nm size by the DLS (Fig. 2E). The SEM and DLS analyses confirmed that even after PTX loading, the CPP modified NPs could retain their size in the optimum range for effective drug delivery (Fig. S1). The zeta values were almost same except for the PTX:R9:Mn:ZnS NPs which could be due to their better PTX loading (Fig. 2F). The PL (photo luminescence) studies confirmed obvious changes in PL intensity before and after CPP modification(Supplementary Fig. S2E–H and M–P). The PL intensity increased significantly higher with the R9 modified NPs among the samples (Supplementary Fig. S3A). The CPP binding efficiency on the Mn:ZnS NPs was maximum for PEN (~80%), followed by pVEC (~40%) and R9 (~20%) respectively (Fig. 2G). On the other hand, PTX encapsulation efficiency (EE) was ~100% with R9 modified NPs followed by Mn:ZnS NPs (~82%), PEN (~53%), pVEC (~20%).(Fig. 2H) The possible PTX interaction with CPP modified NPs could be via the anchoring mechanism (Supplementary Fig. S3B)^20^.

### *In vitro* cyto-compatibility and cellular trafficking of CPP coated Mn:ZnS NPs

**Figure 3:**
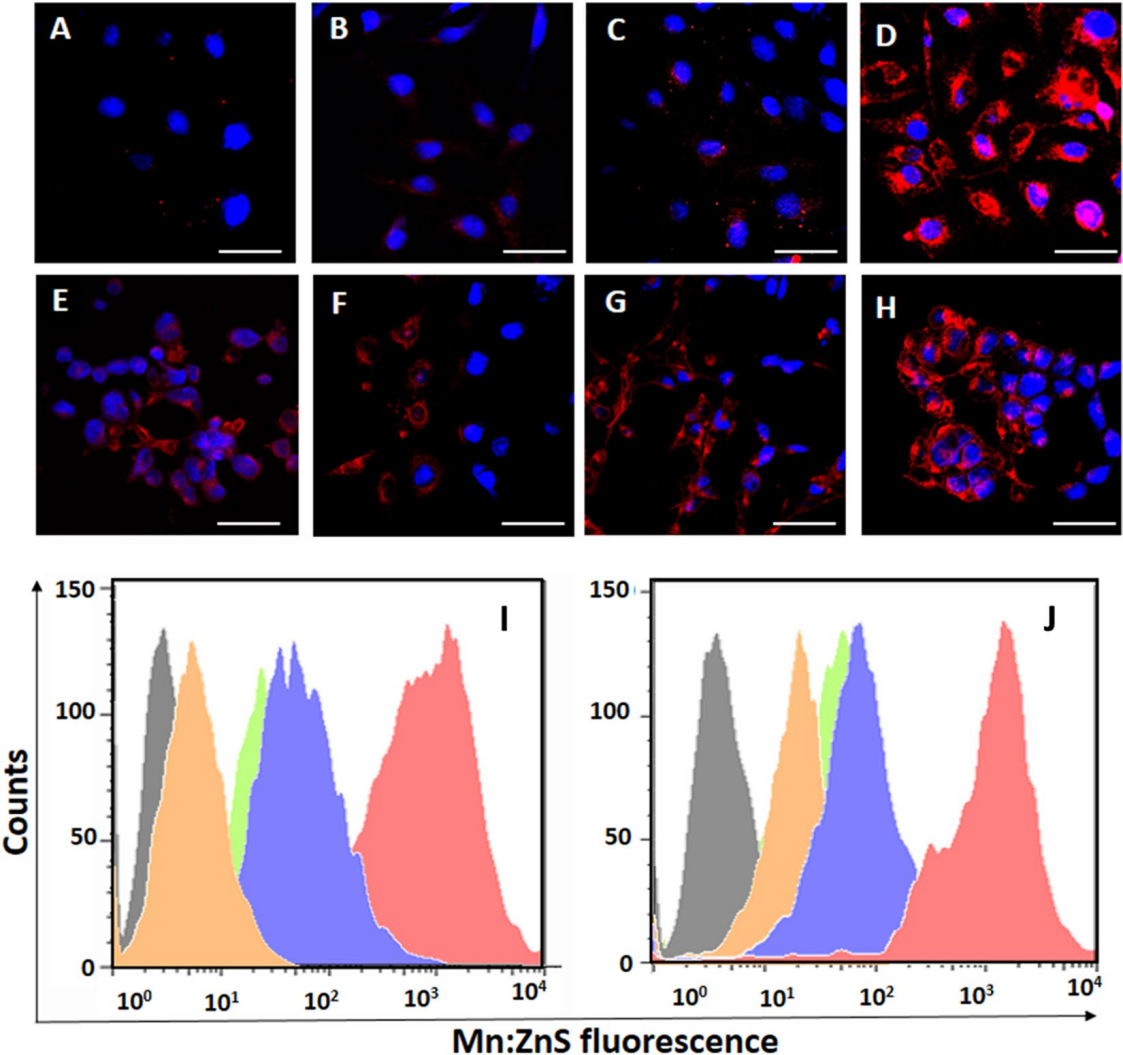
Confocal laser scanning electron microscopic images showing cellular localization pattern of CPP modified NPs: (**A**) Mn:ZnS NPs; (**B**) PEN:Mn:ZnS NPs; (**C**) pVEC:Mn:ZnS NPs; (**D**) R9:Mn: ZnS NPs in SKOV-3 after 24 h incubation period; (**E**) to (**H**) are the same experiments in HeLa cells; (**I**) to (**J**) are their flowcytometry based uptake of CPP modified NPs on SKOV-3 and HeLa cells, respectively, after a 24 incubation period; The gray histogram represents the control cells, orange histogram for control Mn:ZnS NPs; green histogram for PEN:Mn:ZnS NPs; blue histogram for pVEC:Mn:ZnS NPs; and red histogram for R9:Mn:ZnS NPs (Red emission indicates the fluorescence from Mn:ZnS NPs, and blue represents the DAPI nuclear stain’s florescence, scale bar 50 µm; the bright reddish pink color indicates the co-localization of DAPI and Mn:ZnS NPs).

As shown in Fig. S4A,B, the bare samples were compatible on the tested cell lines. The MTT results were then validated with a live/dead assay showing no red fluorescence in all the treated cells (Fig. S4) indicating non toxicity. As shown in Fig. 3, it was clear that CPP modification resulted in enhanced fluorescence within the cells. The CPP modified NPs were well taken up by the SKOV-3 cells without changing the integrity of the nucleus, as the DAPI florescence was well retained in nucleus. The intracellular fluorescence was improved with R9 modified NPs (Fig. 3D,H) compared to the PEN and pVEC which was confirmed by flow cytometry (Fig. 3I,J).

### *In vitro* PTX release

It was done in two different pHs (5 and 7.4). The pH 5 was selected as it is the endosomal pH^21^ whereas pH 7.4 is the normal pH of the blood stream. As shown in the Supplementary Fig. S5, the PTX was released much faster in pH 5 within 48 h, whereas under the neutral pH, the release was extended upto 2 days resulting in 100% PTX release. The PEN:Mn:ZnS NPs have shown slow release and ~80% release was there within 7 days in acidic pH, while ~40% release was there in 7 days under pH 7.4. The pVEC:Mn:ZnS NPs had fast PTX release (almost 100%) within 3 h at pH 5, whereas the neutral pH extended the release 3 days till 100%. On the other hand, the PTX was released slowly from R9 modified NPs, with only ~60% release at pH5 in 7 days. The release % was significantly low as ~30% under neutral condition after 7 days suggesting that the R9 could release PTX more controllably.

### Improved anticancer efficacy with CPP functionalization

**Figure 4:**
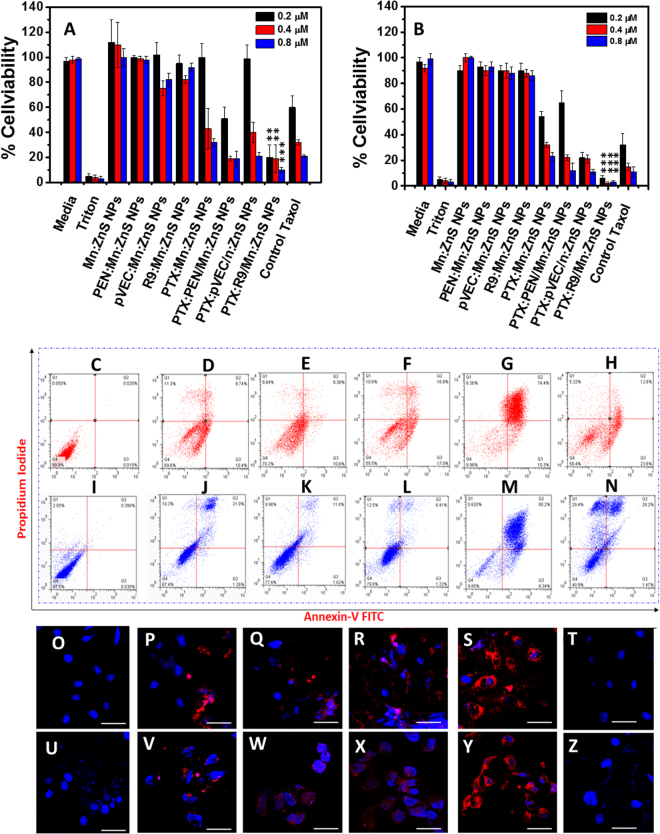
*In vitro* therapeutic efficacy assessment on (**A**) SKOV-3 and (**B**) HeLa cells by PTX-CPP modified Mn:ZnS NPs after 48 h incubation; The control vehicle concentration is 50, 100, and 200 µg/mL, whereas the control PTX concentration is 10, 20 and 30 µM. The actual PTX concentration in samples are 0.2, 0.4 and 0.8 µM respectively. (n = 6, **represents p < 0.01, ***represents p < 0.001); (**C**–**N**) represents the apoptotic profile on SKOV-3 and HeLa cells by the developed PTX formulations with CPP modified Mn:ZnS NPs: (**C**) Control SKOV-3 cells; (**D**) Mn:ZnS NPs; (**E**) PEN/Mn:ZnS NPs; (**F**) pVEC/Mn:ZnS NPs; (**G**) R9/Mn:ZnS NPs and (**H**) Control PTX; (**I**) control HeLa; (**J**) Mn:ZnS NPs; (**K**) PEN/Mn:ZnS NPs; (*L*) pVEC/Mn:ZnS NPs; (**M**) R9/Mn: ZnS NPs and (**N**) Control PTX cells after 48 h incubation time. (**O**–**Z**) shows the confocal laser scanning electron microscopic images on SKOV3 and HeLa cells: (**O**) control SKOV-3; (**U**) Control HeLa cells; (**P**,**V**) PTX:Mn:ZnS NPs; (**Q**,**W**) PTX:PEN/Mn:ZnS NPs; (**R**,**X**) PTX/pVEC:Mn:ZnS NPs; (**S**,**Y**) PTX:R9/Mn:ZnS NPs and (**T**,**Z**) Control PTX respectively after 48 h incubation time. Blue fluorescence is DAPI staining on the nucleus and red fluorescence indicates the Mn:ZnS NPs (scale bar = 50 µm).

The MTT assay was conducted with PTX:CPP modified NPs with concentration ranging from 50 to 200 µg/mL, where the concentration of PTX was maintained at 0.2, 0.4, and 0.8 µM. PTX:R9:Mn:ZnS NPs induced higher toxicity both in SKOV-3 and HeLa cells (Fig. 4A,B) than the other CPP:Mn:ZnS NPs with a minimum concentration of 0.2 µM compared to the control PTX (10 µM). However, the higher concentrations of the control CPP modified NPs did not induce any toxicity up to 200 µg/mL, suggesting that the induced toxicity could be merely due to the internalized PTX-CPP modified NPs. The MTT results were further validated with a live/dead assay, as shown in Supplementary Fig. S6.

The apoptotic % was higher in all the PTX:CPP:Mn:ZnS NPs with maximum % in R9 modified NPs, as shown in Fig. 4G. The PTX:Mn:ZnS NPs had ~ 30% apoptosis (Fig. 4D,J), ~28% for PTX:PEN modified NPs (Fig. 4E,K), ~44% for PTX:pVEC (Fig. 4F,L) and 90% for the PTX:R9 NPs respectively (Fig. 4G,M) NPs. The control PTX showed ~40% apoptotic cells (SKOV-3), possibly due to their less uptake in SKOV-3 cells. The control samples were non apoptotic (Fig. S7). HeLa cells had also smilar results (Fig. S8). These results were confirmed by confocal analysis where the PTX:R9 NPs showed maximum apoptotic features. (Fig. 4S,Y). These reults were further confirmed by a DNA fragmentation analysis (Supplementary Fig. S9A1 and B1). MTT on CT26, B16F10, and 4T1 were done as we planned *in vivo* efficacy and bio distribution analysis on 4T1 Xenograft model (Supplementary Figs S10–S12).

### Long term serum stability of NPs

**Figure 5:**
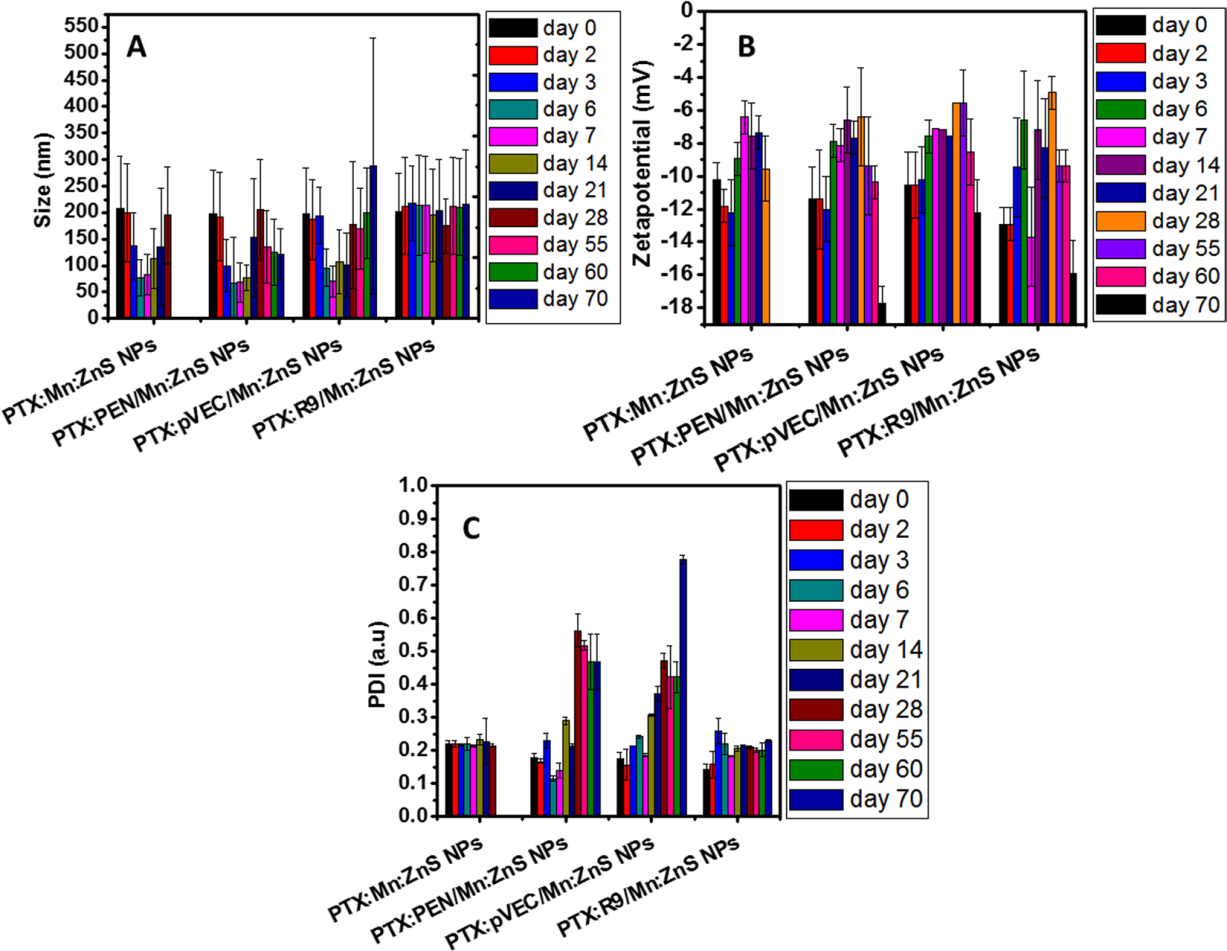
Long-term stability assessment of various PTX:CPP:NPs in 10% FBS at 37 °C for 70 days: (**A**) DLS analysis; (**B**) Zeta potential and (**C**) PDI values (n = 3).

The stability of the samples were well retained, as there was bright fluorescence from the samples (Supplementary Fig. S13). This could be associated with the effective stabilization of CPP coating on PTX:Mn:ZnS NPs. However, control PTX:Mn:ZnS NPs started degrading after day 21(Fig. 5A,B), and colloidal stability indices were not in detection range (Fig. 5C), indicating their poor serum stability.

### *In vivo* biodistribution and preclinical therapeutic evaluation using 4T1 breast tumor xenograft model

**Figure 6:**
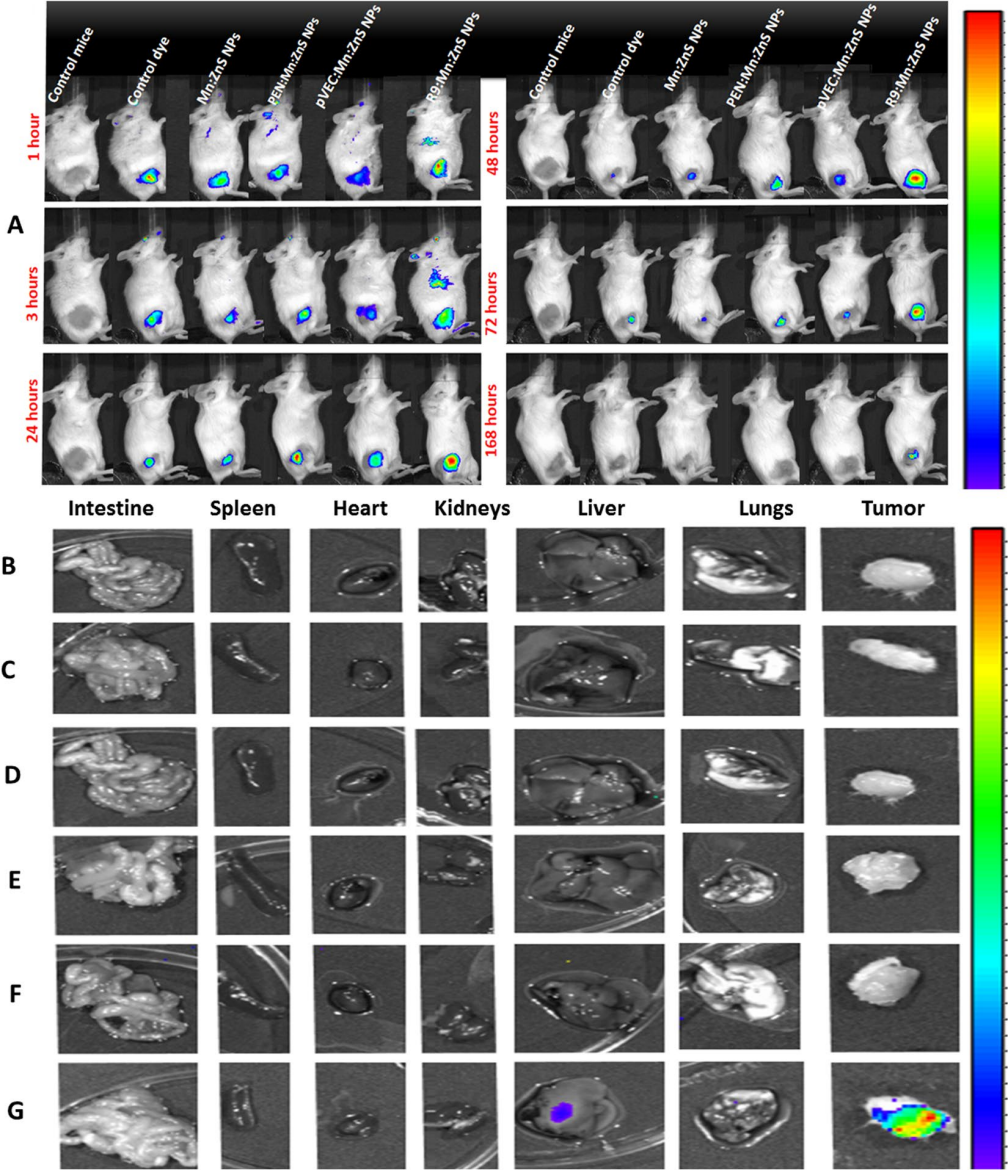
Bio-distribution analysis of IR-780 doped samples on 4T1 tumor model. Left to right samples: Control mice, control dye, IR-780:Mn:ZnS NPs, IR-780:PEN/Mn:ZnS NPs, IR-780:pVEC/Mn:ZnS NPs, IR-780:R9/Mn:ZnS NPs respectively from 1 hour to 168 h. (**B**–**G**) shows tumor vs organ *ex vivo* bio-imaging on 4T1 tumor model one week after i.v injection. (**B**) Control mice; (**C**) control dye; (**D**) IR-780:Mn:ZnS NPs; (**E**) IR-780:PEN/Mn:ZnS NPs, (**F**) IR-780:pVEC/Mn:ZnS NPs, (**G**)IR-780:R9/Mn:ZnS NPs respectively.

The IR-780 modified NPs showed similar particle size with unmodified NPs (Supplementary Fig. S14). The fluorescence intensity of IR-780 loaded Mn:ZnS NPs was much higher than control IR-780 (Supplementary Fig. S15A and B). Compared to the IR-780 dye, (which retained only for 3 days after i.v. injection), the R9/Mn:ZnS NPs showed high retention in tumor even after 7 days. (Fig. 6A) This was confirmed by *ex vivo* imaging. (Fig. 6B–G)

**Figure 7:**
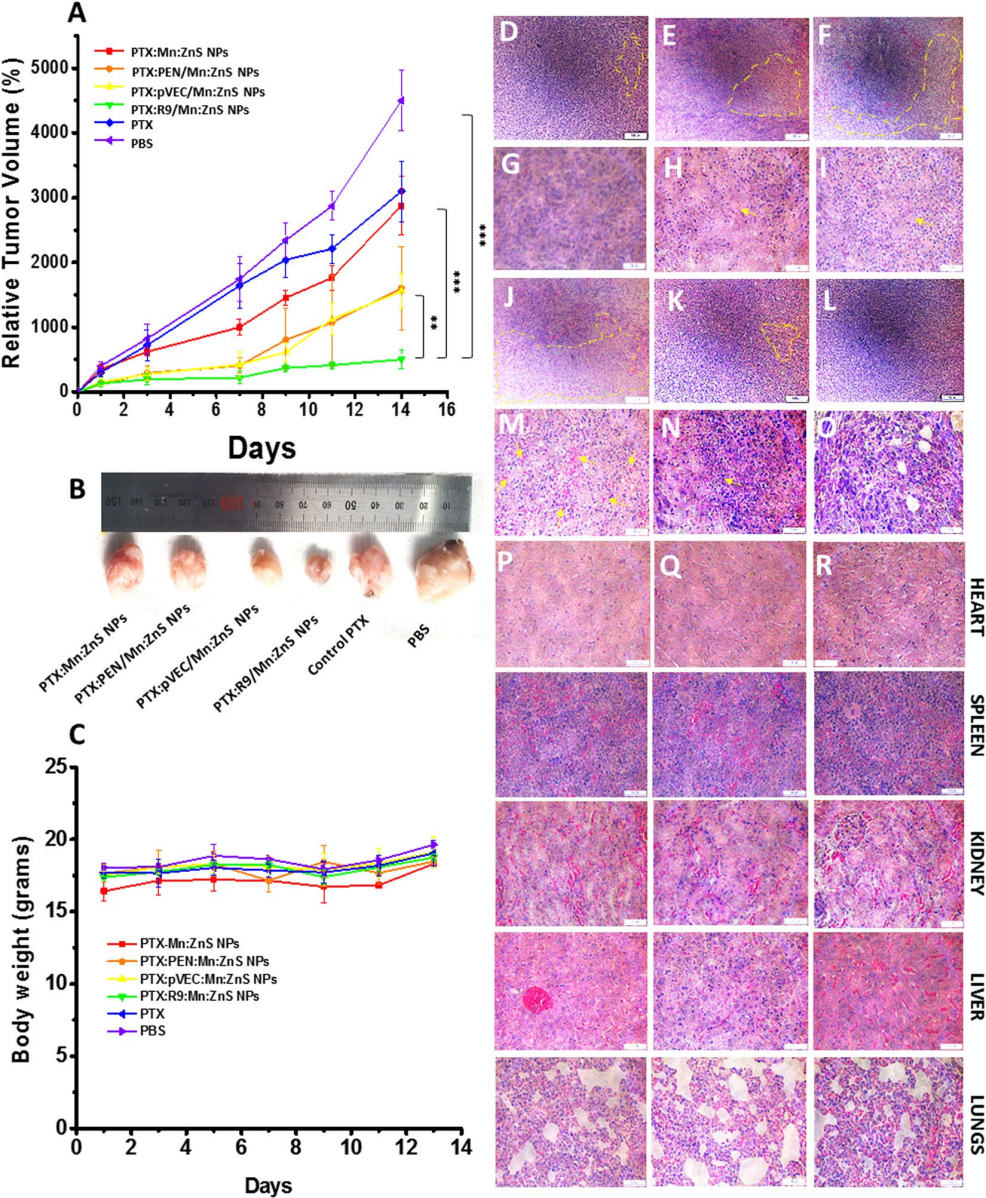
Therapeutic efficacy studies using breast tumor xenograft model (Balb/c mice): (**A**) tumor reduction studies after injecting the samples once per week till 14 days; (**B**) represents the tumor tissues dissected two weeks after the therapy, showing significant reduction with PTX:R9/Mn:ZnS NPs compared to the other CPP modified PTX loaded Mn:ZnS NPs. (**C**) shows no body weight loss after treatment with the respective sample for two weeks of experiments. (**D**–**O**) represents the (**H**,**E**) staining of tumors two weeks after euthanasia, showing apoptotic pathology. (**D**–**F**,**J**–**L**) represents the PTX:Mn:ZnS NPs, PTX:PEN:Mn:ZnS NPs, PTX: pVEC/Mn:ZnSNPs,PTX:R9/Mn:ZnS NPs; control PTX, PBS (lower magnification of 10×) and (**G**–**I**,**M**–**O**) shows their higher magnifications (40×) respectively; The yellow dots show the affected area and arrows represent the apoptotic nuclei. (**P**–**R**) are the **H and** **E** stained major organ tissues after treatment with (**P**) PTX:R9/Mn:ZnS NPs; (**Q**) control PTX, (**R**) PBS showing non-toxicity at *in vivo* ((n = 6, **represents p < 0.01 and ***represents p < 0.001).

*In vivo* efficacy studies revealed maximum tumor efficacy for PTX:R9:Mn:ZnS NPs (Fig. 7A,B) as confirmed by H & E staining showing maximum apoptotic pathology (Fig. 7M) followed by PTX:PEN/Mn:ZnS NPs, PTX:pVEC/Mn:ZnS NPs, PTX:Mn:ZnS NPs and PTX formulation. The body weight of all animals were maintained in healthy range indicating their safety *in vivo* (Fig. 7C). Additionally, as shown in Fig. 7D–O, there were no significant pathological changes in any of the major organs (Fig. 7R). This suggests that these formulations might be good candidates for bio-applications. Further, Fig. 7R clearly indicates that the treatment did not affect the body weight of animals, assuring nontoxic behavior, as observed in the H and E staining studies.

## Discussion

Optimum particle size is important for efficient drug delivery applications^22^. All the synthesized CPP modified Mn:ZnS NPs showed spherical shapes and the possible interaction could be via the metal-polymer interaction^23^. The CPP modified Mn:ZnS NPs showed improved fluorescence as well. The high fluorescence with the PTX loaded Mn:ZnS NPs might be due to the higher anchoring of PTX on the Mn:ZnS core(Supplementary Fig. S3B). This could lend Mn:ZnS NPs greater stability, thereby high fluorescence among the other samples^24^. The PTX anchoring could actually favor surface passivation of the Mn:ZnS NPs to have better fluorescence^25^. The high PTX EE of R9 with low surface passivation capability could be attributed to strong metal:polymer interaction with the PTX molecules. In addition, the exposed core in the case of R9/Mn:ZnS NPs could additionally favor anchoring of PTX onto it, giving more space for PTX binding.

The interaction between CPPs and Mn:ZnS could be either via the strong metal to ligand interaction or by the electrostatic interaction between Mn:ZnS and CPPs. It is known that the amino acid sequence in CPPs is electron rich, allowing donation of electrons to the vacant d-shells in the Mn:ZnS leading to strong bonding between them^26^. The difference in the CPP binding on the Mn:ZnS could be attributed to the difference in amino acid sequence and electron density in it. The PEN has amino acid sequence of RQIKIWFQNRRMKWKK, which may have enhanced electron density, and thus the interaction would be higher than that of pVEC and R9, respectively.

The cytocompatibility as well as the cellular assessment showed that the developed formulations were nontoxic, thus favoring applications for *in vivo* animal studies. The higher uptake of R9 modified NPs in all the tested cell lines could be attributed to their arginine abundant amino acid sequence, which could enable selective entry into cancer cells^27^. The improved anti-cancer efficacy of the PTX:R9:Mn:ZnS NPs could be due to the higher intracellular localization capability. In the case of PTX, it is highly recommended to have an intracellular availability of PTX to show its potency against cancer cells. PTX is one of several cytoskeletal drugs that can specifically target tubulin. PTX can affect the mitotic spindle assembly, chromosome segregation, and cell division^28^. The R9:Mn:ZnS NPs has benefits of maximum PTX loading capability (Supplementary Table S1) which might be enhanced in an acidic environment, lending higher toxicity to the treated cancer cells. The *in vitro* PTX release showed controlled and sustained release from R9:Mn:ZnS NPs which could be related to its high anti-tumor efficacy *in vivo*. Additionaly the doped ZnS NPs could increase the ROS generation thereby improving the apoptosis along with the PTX^7^. Unlike the other CPP modified samples, the PTX:R9:Mn:ZnS NPs showed a very different stability pattern, retaining stability even up to 70 days. The particle size and colloidal satiability indices were maintained throughout the 70 days, as presented in Fig. 5aA–C. This high stability could assure its *in vivo* therapeutic efficacy than other samples.

*In vivo* live imaging is critical for drug delivery systems.Unlike the undoped ZnS NPs, Mn:ZnS NPs has many advantages such as improved cytocompatibility, enhanced ROS generation etc, however it cannot be used for live imaging as its fluorescence is in the UV range. Obviously the tissue penetration of UV light is very poor compared to the NIR light^29^. Infact practical use of UV light for live imaging is hazardous as UV radiation is classified as a “complete carcinogen” because it is both a mutagen and a non-specific damaging agent and has properties of both a tumor initiator and a tumor promoter^30^. Thus with the inherent fluorescence of Mn:ZnS NPs, it is difficult to do the live imaging in the animal models. Therefore we used IR-780 dye which was doped in the NPs where it can stay well in the space lattice of NPs without undergoing any degradation or leaching. Additionally, since the IR-780 is doped in the core material there would be minimum interaction with the co-laoded PTX as they are attached to the CPP shell. This was seen in the bio distribution study as most of the R9:Mn:ZnS NPs CPP could retain in the tumor even after a week than other samples. This could be associated with its enriched arginine composition that facilitate intratumoral entry^31^. Since PTX has poor solubility, the R9:Mn:ZnS NPs may enhance its tumor localization to improve efficacy. In addition, the R9 coated Mn:ZnS NPs were nontoxic to the internal organs as evident in the histopathology analysis (Fig. 7P). The enhanced *in vivo* theranostic property of PTX:R9:Mn:ZnS NPs could be due to the better tumor localization capability, improved ROS generation thereby better apoptois along with the PTX effect. Eventhough there have been few reports regarding the applicability of Mn:ZnS NPs for drug delivery, most of them are confined to *in vitro* studies without any pre-cliniacl analysis on animal models. The studies by Yu *et al*., (2013) showed that their Mn:ZnS NPs could only be attached on the surface of the tested cells than being taken up inside the cells which might reduce the therapeutic outcome^32^. Another study by Zhao *et al*., (2017) used PTX loaded Mn:ZnS NPs lacks the therapeutic analysis^12^. To the best of our knowledge no paper has been reported with CPP functionalization for IR-780:Mn:ZnS NPs to have simultaneous imaging and therapy, thus our current study could open up new channels for using IR-780 doped Mn:ZnS NPs for cancer drug delivery appications.

## Conclusions

Different CPP coated Mn: ZnS NPs were made to evaluate their potency as drug delivery and imaging agents against cancer cells. The size of the developed NPs would be ideal for *in vivo* applications. The *in vitro* studies including PL spectroscopy suggest that the fluorescence has been improved with the CPP modification, and the NPs were well localized in all the tested cell lines. Among the CPP modified PTX encapsulated NPs, the R9 modified PTX loaded NPs were found to be more cytotoxic to the cancer cells, which has been proven by MTT, live/dead, DNA fragmentation, and apoptosis assay. The bio-distribution analysis showed that the R9 modified IR-780 doped Mn:ZnS NPs accumulated significantly higher than other CPP modified NPs, which was validated with *ex vivo* imaging. The *in vivo* analysis showed highest anticancer potency for PTX: R9/Mn:ZnS NPs compared to the other samples without any toxicity to the internal organs. These preliminary results suggest that the PTX:R9/Mn:ZnS NPs may be a better PTX delivery agent against cancer cells, compared to the other CPP modified NPs.

## Materials and Methods

### Materials

CPPs (PEN, pVEC, R9, and their FITC modified versions) were custom made by Peptron Company, Republic of Korea. ZnCl_2_, MnSO_4_ were purchased from Junsei Chemical Co. Limited, South Korea. Na_2_S was purchased from Sigma Aldrich, USA.

### Synthesis of Mn:ZnS and surface passivation with CPPs (PEN, pVEC and R9)

The Mn:ZnS NPs were synthesized according to previous protocols with slight modifications^8^. Briefly, 0.1 M ZnCl_2_ was made in 4 mL of distilled water (pH was adjusted to 5, inorder to dissolve ZnCl_2_ properly) and then mixed with an equal volume of 0.01 M MnSO_4_ solution (pH 7). To this solution, a 0.1 M Na_2_S was added drop-wise under continuous probe sonication (~2 min) untill there was white opalescent coloration (indicative of Mn:ZnS NPs formation) followed by two times centrifugation at 13,000 rpm for 30 min, to separate remnant solutes from the NPs. For surface passivation with CPPs, the CPPs were made at 0.01 mM concentration. Each of the CPPs were directly added into the preformed Mn:ZnS NPs (1 mg/mL) and surface passivated for about 30 min followed by centrifugation at 13,000 rpm (30 min) to remove the unwanted solutes.

### CPP binding efficiency of modified Mn:ZnS NPs

Detailed protocls have been given in File S1.

### Synthesis of PTX Loaded CPP Modified Mn:ZnS NPs, encapsulation efficiency, and *in vitro* drug release

Briefly, 3.6 µM PTX solution was added to the mixture of 0.1 M ZnCl_2_ and 0.01 M MnSO_4_ aqueous solution(pH 5) followed by the addition of 0.1 M Na_2_S under continuous probe sonication for about 2 min. Different CPPs were then added to coat it. The solutions were stirred under 600 rpm for 10 min, centrifuged two times, washed and centrifuged at 13,000 rpm for 30 min. The final suspensions were dispersed in distilled water.The detailed protocol for PTX release studies have been given in Supplementary File S2.

### Synthesis of IR-780 Doped-Mn:ZnS and CPPs (PEN, pVEC and R9) modified Mn:ZnS NPs

The IR-780 dye was doped into Mn:ZnS NPs and further coated with CPP by a two-step protocol. Detailed protocol has been given in Supplementary File S3. The IR-780 dye was used mainly to trace the *in vivo* biodistribution of the Mn:ZnS NPs. Since Mn:ZnS NPs has IR absorption, it would be difficult to trace them by its innate fluorescence, whereas the IR-780 dye doped Mn:ZnS NPs would be highly beneficial for *in vivo* NIR imaging.

### Evaluation of cytocompatibility, cellular localization using confocal microscopy, flowcytometry, and optical microscopy

Refer Supplementary File S4 for detailed experimental protocols.

### *In vitro* anti-cancer efficacy of CPP modified Mn:ZnS NPs on SKOV-3 and HeLa cells

Detailed protocol has been given in Supplementary File S5.

### Long term stability studies of NPs

10% FBS was used for this. The samples were made as per the literature^33,34^. Precisely, 0.5 mL samples were mixed with 1 mL of 10% FBS, and incubated at 37 °C for predetermined time intervals. The samples were collected after each time for DLS/Zeta/PDI determination.

### *In vivo* studies

All animal experimental protocols and methods complied with the principles of Laboratory and Animal Care established by the National Society for Medical Research and were approved by the Korea Advanced Institute of Science and Technology (KAIST) on Use and Care of Animals. Details of *in vivo* studies have been given in Supplementary File S6. The protocols for Histopathology was done according to  the previous work^35,36^.

### Analytical determinations

Refer Supplementary File S7.

### Statistical analysis

Statistical significance of differences values were calculated by a two-tailed Student’s t-test. For multiple comparisons, an ANOVA analysis was performed. A value P < 0.05 was considered to be statistically significant.

## Electronic supplementary material

Supplementary Information
