# Author Correction: Evaluation of cell penetrating peptide coated Mn:ZnS nanoparticles for paclitaxel delivery to cancer cells

**DOI:** 10.1038/s41598-018-31646-5

**Published:** 2018-09-04

**Authors:** N. Sanoj Rejinold, Yunho Han, Jisang Yoo, Hae Yong Seok, Ji Ho Park, Yeu-Chun Kim

**Affiliations:** 1 Department of Chemical and Biomolecular Engineering, Korea Advanced Institute of Science and Technology (KAIST), Daejeon, Republic of Korea; 2 Department of Brain and Bioengineering, Institute of Health Science and Technology, Korea Advanced Institute of Science and Technology (KAIST), Daejeon, Republic of Korea

Correction to: *Scientific Reports* 10.1038/s41598-018-20255-x, published online 30 January 2018

In this Article, Figure 4O is a duplication of Figure 3B. The correct Figure 4 appears below as Figure 1.

**Figure 1:**
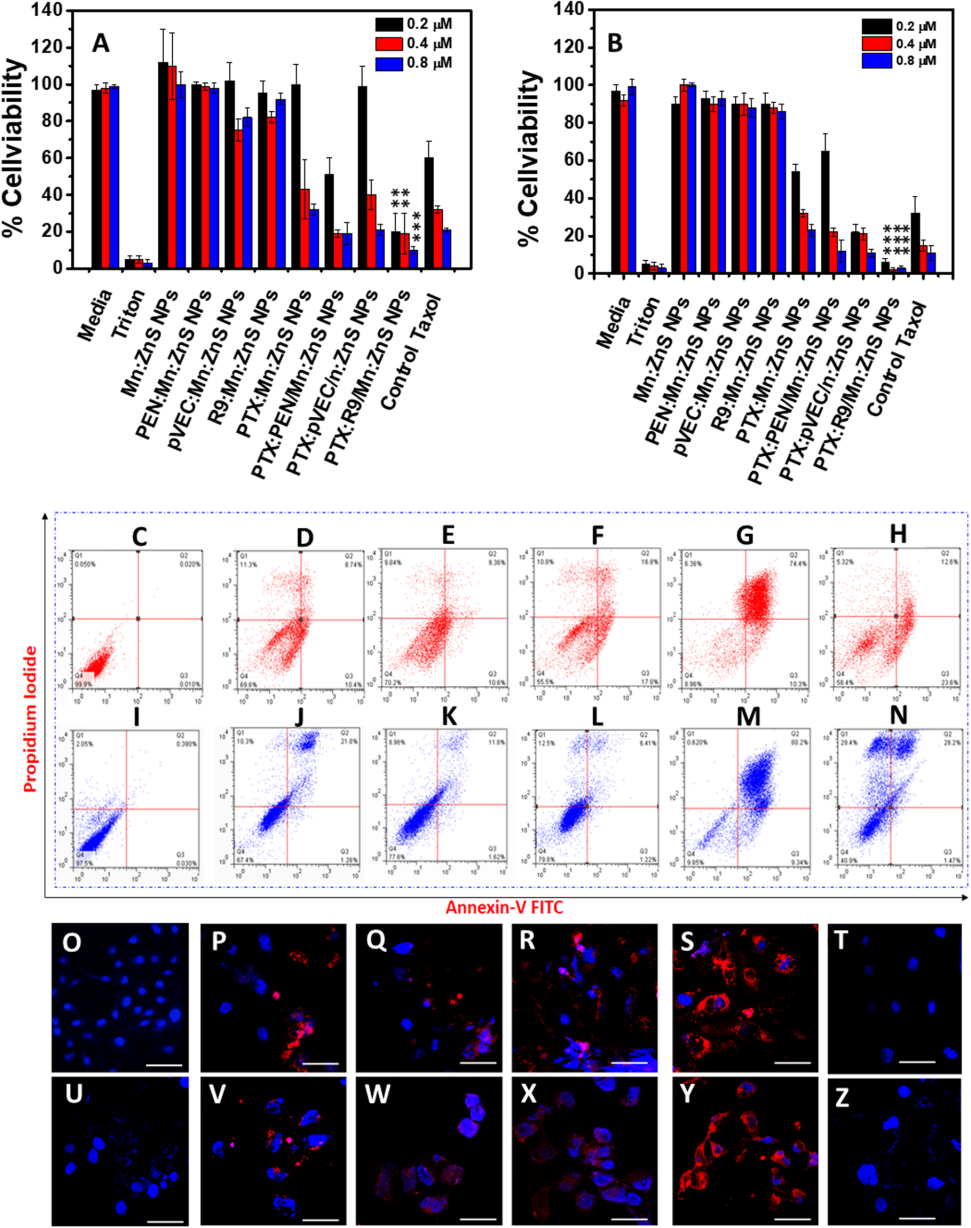
*In vitro* therapeutic efficacy assessment on (**A**) SKOV-3 and (**B**) HeLa cells by PTX-CPP modified Mn:ZnS NPs after 48 h incubation; The control vehicle concentration is 50, 100, and 200 μg/mL, whereas the control PTX concentration is 10, 20 and 30 μM. The actual PTX concentration in samples are 0.2, 0.4 and 0.8 μM respectively. (*n* = *6*, **represents p < *0*.*01*, ***represents p < *0.001*); (**C**–**N**) represents the apoptotic profile on SKOV-3 and HeLa cells by the developed PTX formulations with CPP modified Mn:ZnS NPs: (**C**) Control SKOV-3 cells; (**D**) Mn:ZnS NPs; (**E**) PEN/Mn:ZnS NPs; (**F**) pVEC/Mn:ZnS NPs; (**G**) R9/Mn:ZnS NPs and (**H**) Control PTX; (**I**) control HeLa; (**J**) Mn:ZnS NPs; (**K**) PEN/Mn:ZnS NPs; (**L**) pVEC/Mn:ZnS NPs; (**M**) R9/Mn: ZnS NPs and (**N**) Control PTX cells after 48 h incubation time. (**O**–**Z**) Shows the confocal laser scanning electron microscopic images on SKOV3 and HeLa cells: (**O**) control SKOV-3; (**U**) Control HeLa cells; (**P**,**V**) PTX:Mn:ZnS NPs; (**Q**,**W**) PTX:PEN/Mn:ZnS NPs; (**R**,**X**) PTX/pVEC:Mn:ZnS NPs; (**S**,**Y**) PTX:R9/Mn:ZnS NPs and (**T**,**Z**) Control PTX respectively after 48 h incubation time. Blue fluorescence is DAPI staining on the nucleus and red fluorescence indicates the Mn:ZnS NPs (scale bar = 50 μm).

In addition, Figure 6B is a duplication of Figure 6D. The correct Figure 6 appears below as Figure 2.

**Figure 2:**
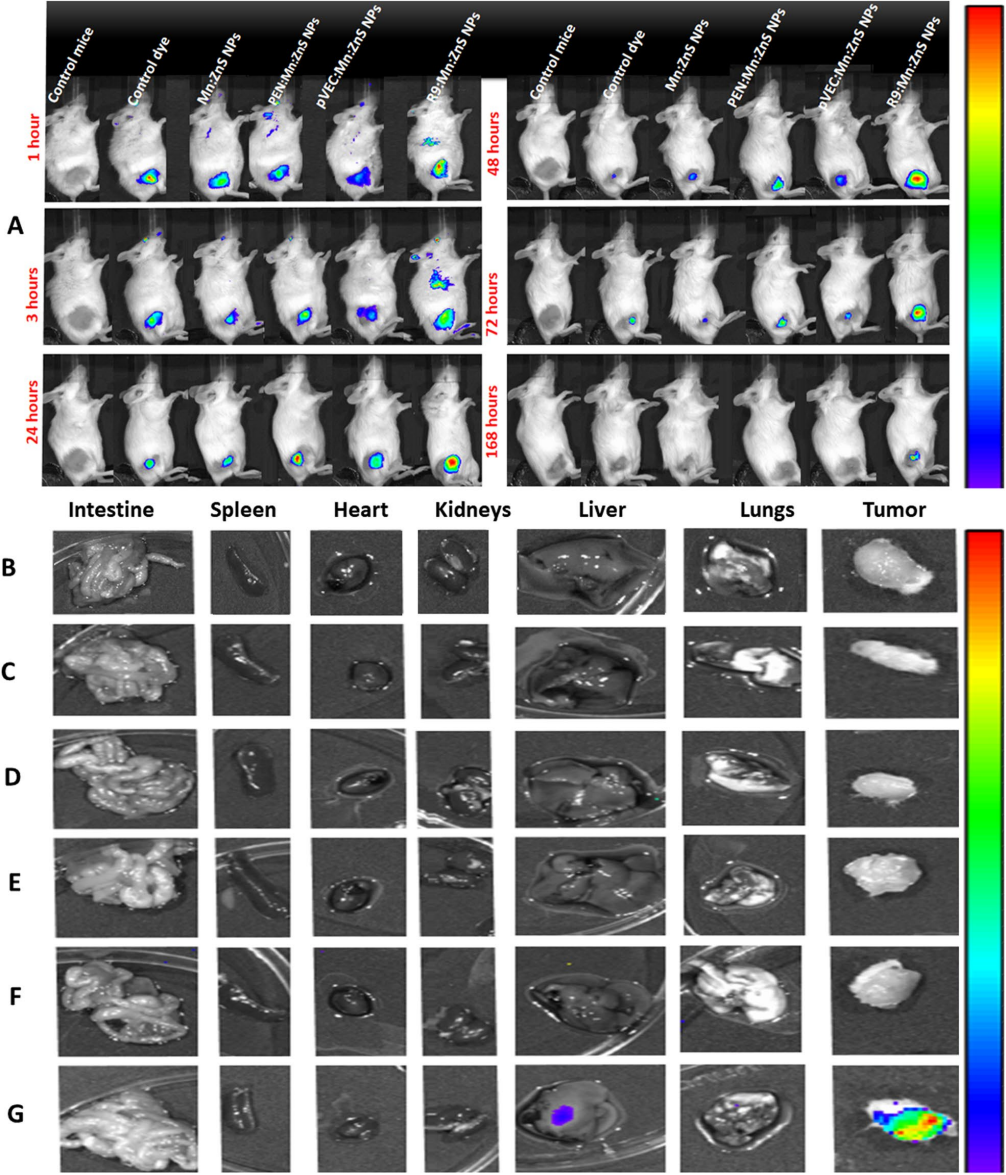
Bio-distribution analysis of IR-780 doped samples on 4T1 tumor model. Left to right samples: Control mice, control dye, IR-780:Mn:ZnS NPs, IR-780:PEN/Mn:ZnS NPs, IR-780:pVEC/Mn:ZnS NPs, IR-780:R9/Mn:ZnS NPs respectively from 1 hour to 168 h. (**B**–**G**) shows tumor vs organ *ex vivo* bio-imaging on 4T1 tumor model one week after i.v injection. (**B**) Control mice; (**C**) control dye; (**D**) IR-780:Mn:ZnS NPs; (**E**) IR-780:PEN/Mn:ZnS NPs, (**F**) IR-780:pVEC/Mn:ZnS NPs, (**G**) IR-780:R9/Mn:ZnS NPs respectively.

