# Supplementary material for: Evaluation of cell penetrating peptide coated Mn:ZnS nanoparticles for paclitaxel delivery to cancer cells
Source: Sci Rep. 2018 Jan 30;8:1899. doi: 10.1038/s41598-018-20255-x (PMC5789852; doi:10.1038/s41598-018-20255-x)
Supplement: Supplementary file 1 — Supplementary Information [file 41598_2018_20255_MOESM1_ESM.doc]

**Supplementary Information**

Evaluation of cell penetrating peptide coated Mn:ZnS nanoparticles for paclitaxel delivery to cancer cells

N. Sanoj Rejinold^1^, Yunho Han^2^, Jisang Yoo^1^, Hae Yong Seok^1^, Ji Ho Park^2^ & Yeu-Chun Kim^1*^

^1^ Department of Chemical and Biomolecular Engineering, Korea Advanced Institute of Science and Technology (KAIST); Daejeon, Republic of Korea; Tel: 82-42-350-3939; Fax: 82-42-350-3910

^2^Department of Brain and Bioengineering, Institute of Health Science and Technology, Korea Advanced Institute of Science and Technology (KAIST); Daejeon, Republic of Korea; Tel: 82-42-350-3939; Fax: 82-42-350-3910

***Corresponding author’s e-mail:** **dohnanyi@kaist.ac.kr**;

**Tel: +82-42-350-3939; Fax: +82-42-350-3910**

***File S1.*** ***CPP Binding Efficiency of Modified Mn: ZNS NPs***

The CPP surface passivation capability was analyzed using photoluminescence spectroscopy. For this the FITC-labelled CPPs (Peptron, South Korea Made) were used. Briefly, the FITC labelled CPPs were modified with the Mn: ZnS NPs as described above and the final solutions were analyzed to determine the amount of CPP attached on the surface of the Mn: ZnS NPs. R9-Biotin conjugate was used for detecting the surface passivation of R9 on Mn:ZnS NPs. Briefly, 0.01 mM R9-Biotin was incubated with a mixture of 0.1 M MnSO_4_ and 0.01 M ZnCl_2_ solution under mild probe sonication. After 30 min, the solution was treated with the Biotin-R9 solution, as mentioned above. The solution was centrifuged two times and washed, and then 0.001 M streptavidin solution was added to obtain the green fluorescent final product, which was quantified by using photoluminescence spectroscopy.

***File S2.*** *Encapsulation efficiency and in vitro drug release studies*

The encapsulation efficiency was determined by UV spectroscopy (Optizen 3220 UV) using a reported method.^1^ For *in vitro* drug release studies, we adapted direct dispersion method^2^, in which the samples were dispersed in phosphate buffered saline (pH 5 and 7.4). The 7.5 mL CPP modified Mn: ZnS NPs (1mg/mL in PBS 7.4) were equally distributed in 30 eppendorf tubes by aliquoting 250µL of the samples. Similarly, another set of samples were prepared in PBS having pH 5. These samples were kept at shaking incubator for seven days. At predetermined time intervals starting from 1, 3, 6, 24, 48, 72, 96 and 168 h, the samples were withdrawn and centrifuged at 13,000 rpm for 10 min to separate the released PTX from the formulations. The released PTX was further analyzed by using UV spectrometer at 220 nm.

***File S3. Synthesis of NIR-780 doped-Mn: ZnS and CPPs (PEN, pVEC and R9) modified Mn:ZnS NPs***

The IR-780 dye was doped into Mn: ZnS NPs and the CPP modified nanostructures by a two-step protocol. In the first step, 0.1 M ZnCl_2_ was prepared in 4 mL of distilled water as mentioned before and then mixed with an equal volume of 0.01 M MnSO_4_ aqueous solution. In the second step, 200 µL of 0.1 mg/mL of ethanolic NIR-780 dye and 0.1 M Na_2_S was added drop-wise under continuous probe sonication for about 2 min. The NIR-780 dye loses it color when it was mixed with Na_2_S, whereas the color immediately re-appeared when it is doped into the Mn:ZnS NPs. The probe sonication was continued till there was a white opalescent coloration, indicative of the formation of Mn: ZnS NPs (~ 1 min probe sonication at 10% amplitude) which were further coated with different CPP molecules as described before. These samples were then centrifuged at 13,000 rpm for about 30 min, to separate the NPs. The centrifugation steps were repeated twice to wash off all the unreacted solutes.

***File S4. Evaluation of cytocompatibility, Cellular localization using Confocal microscopy, flowcytometry, and Optical microscopy***

SKOV-3 (Human ovarian cancer cells) were gifted by the Department of Biological Sciences; KAIST and HeLa (Human cervical cancer cells) were purchased from ATCC, USA. They were maintained in DMEM media supplemented with 10% fetal bovine serum (FBS). The cells were incubated with 5% CO_2_. After reaching confluency, the cells were detached from the flask with trypsin-EDTA. The cell suspension was centrifuged at 3000 rpm for 3 min and then re-suspended in the growth medium for further studies.

***Live/Dead Assay***

Live/dead assay was done according to the previous protocol.^3^ For live/dead assay tests, the cells were seeded on 12-well plates with a seeding density of 20,000 cells. The samples were treated once they reached ~ 80% confluency and assessed for their response with the sample treatment. The PTX loaded CPP modified NPs treated and non-treated cells then stained with a live/dead assay kit and viewed under a fluorescent microscope for the analysis.

***Cytocompatibility***

For compatibility experiments, SKOV-3 and HeLa cells were seeded on a 96 well plate with a density of 10,000cells/cm^2^. Different concentrations of the samples (5 to 200 µg/mL) were prepared by diluting with media. For cellular localization studies, the samples with concentration of 50µg/mL were treated with SKOV-3 and HeLa cells with a seeding density of 5000 cells/well. The cells were harvested after 24h, and washed with PBS twice to remove the non-up taken samples. Since the Mn: ZnS NPs has intense orange/red coloration, we stained the cells with DAPI, which is blue in color, as per the manufacturer’s protocol.

***Cellular localization studies***

As fluorescent microscopy gives a better understanding of the internalization of particles, this was used to confirm the uptake of our samples by the cells. Acid etched cover slips kept in 24-well plates were seeded with SKOV-3 and HeLa cells, respectively, with a seeding density of 2 × 10^4^ cells per cover slip, and they were incubated for 24 h for the cells to attach well. After 24 h incubation, the media were removed and the wells were carefully washed with PBS buffer. The NPs at a concentration of 50µg/mL (mass of NPs, where PTX concentration is 0.2 µM) were then added along with the media in triplicate to the wells and incubated for a time period of 24h. After the given incubation time, the DAPI was added and the cover slips were processed for fluorescent microscopy. The processing involved initially washing the cover slips with PBS and fixing the cells in 3.7% Para Formaldehyde (PFA) followed by a final PBS wash. The cover slips were air dried and mounted onto glass slides with DPX (Sigma Aldrich) as mountant. The slides were then viewed under a confocal microscope (Leica DM2500) to study the internalization of samples. The SKOV-3 and HeLa cells in the log phase were seeded at a density of 50,000 cells/cm^2^ into a 24-well plate for the uptake by flowcytometry. 50µg/mL different samples were made by dilution with the media for this study. After attaining 90% confluency, the cells were washed with PBS buffer, and the above mentioned samples were added and incubated at 37°C for 24 h. Intracellular FITC fluorescence was analysed by flow cytometry after excitation with a 488 nm argon laser using FACS Aria II (Beckton and Dickinson, Sanjose, CA). At 530/30 band pass and 502 long pass, fluorescence emission of 515-545 nm from 10,000 cells was collected, amplified, and scaled to generate a single parameter histogram.

***File S5: In vitro Anti-cancer efficacy of CPP Modified Mn: ZnS NPs on SKOV-3 and HeLa cells***

***a) MTT assay***: For MTT [3-(4,5-Dimethylthiazole-2-yl)-2, 5-diphenyl tetrazolium] assay, the cells were washed with PBS buffer and 50, 100 and 200µg/mL of the NPs (where the PTX concentration was retained as 0.2, 0.4 and 0.8 µM respectively) were added and left for particle incubation of 24h. The sample treated and untreated cells were harvested and analyzed for anti-cancer efficacy of different CPP modified Mn:ZnS NPs. Cells in media alone, devoid of NPs, acted as a negative control and cells treated with Triton X-100 as a positive control for a period of 24 h. 5mg of MTT (Sigma) was dissolved in 1 mL of PBS and filter sterilized. 10 µL of the MTT solution was further diluted to 100 µL with 90 µL of serum–free phenol red free medium. The cells were incubated with 100 µL of the above solution for 4h to form formazan crystals by mitochondrial dehydrogenases. 100 µL of the solubilization buffer (10% Triton X-100, 0.1N HCl and Isopropyl alcohol) was added in each well and incubated at room temperature for 1h to dissolve the formazan crystals. The optical density of the solution was measured at a wavelength of 570 nm using a Thermo-scientific multi-scan go, Finland by a reported protocol. Triplicate samples were analyzed for each experiment.

***b) Apoptosis assay by Flowcytometry***: The phosphatidylserine (PS) translocation from inner to outer layer of the plasma membrane is one of the early apoptotic signs. Thus PS translocation can act as an apoptotic marker for many malignant cells. The PS exposure in SKOV3 and HeLa cells was detected using an Annexin V-FITC/PI Vybrant apoptosis assay kit (Molecular probes, Eugene, OR). The cells were seeded in a 12-well plate with a density of 2x10^4^ / well. After reaching 90% confluency, the cells were treated with different concentrations of Mn: ZnS NPs, PEN/Mn: ZnS NPs, pVEC/Mn: ZnS NPs; R9/Mn: ZnS NPs and their respective PTX loaded versions with control PTX (50µg/mL). After treatment with the samples for about 48 h at 37°C, cells were harvested by trypsinization and washed with PBS for 5 min followed by centrifugation at 500 g at 4°C. The supernatant was discarded and the pellet resuspended in ice-cold 1X Annexin binding buffer (5×10^5^-5×10^6^ cells/mL). 2 μL of Annexin V-FITC solution and 0.5 μL of PI (100μg/mL) were added to 100 μL of the cell suspension and mixed gently. The samples were then incubated at room temperature for 15 min in the dark. After incubation, 400 μL of ice-cold 1X binding buffer was added, mixed gently, and analyzed by flow cytometry( FACS Aria II (Beckton and Dickinson, Sanjose, CA). Cells in media alone devoid of any nanoparticles (negative control) and cells treated with control nanoparticles were also analyzed in the same manner. Samples were analyzed in triplicate for each experiment.

***c) DNA Fragmentation Analysis****:* After treatment with different PTX loaded CPP modified Mn:ZnS NPs ( 50 µg/mL), the HeLa and SKOV-3 cells were harvested and apoptosis was evaluated by an Apoptotic DNA Ladder Kit (Roche Molecular Biochemicals, Mannheim, Germany) according to the manufacturer’s protocol. The DNA solution was separated on 1.2% agarose gel. The samples were loaded onto the gel after 1:5 ratio mixing with the loading star, enabling better resolution of the gel photographs

***File S6: a) In Vivo Bio-distribution Studies****:* All animal experimental protocols and methods complied with the principles of Laboratory and Animal Care established by the National Society for Medical Research and were approved by the Korea Advanced Institute of Science and Technology (KAIST) on Use and Care of Animals. 4T1 murine breast cancer cells were cultured in DMEM (Dulbecco’s Modified Eagle Medium, Hyclone, UT, USA) and supplemented with 10% FBS (Fetal Bovine Serum, Hyclone, UT, USA) and 1% penicillin/streptomycin (Hyclone, UT, USA). All cells were incubated at t 37℃ in 5 % CO_2_. For *in vivo* study, 5 × 10^5^ 4T1 murine breast cancer cells were implanted in the flank of 6 week-old female BALB/c mice. When the volume of tumor reached about 50mm^3^, free dye, PBS, NIR-780:Mn:ZnS NPs; NIR-780:PEN/Mn:ZnS NPs; NIR-780:pVEC/Mn:ZnS NPs; NIR-780:R9/Mn:ZnS NPs were injected intravenously at a dose of 1mg/kg. Animal weight and tumor volume were routinely measured. *In vivo* live imaging followed by *ex vivo* tissue imaging was done using IVIS Lumina (Xenogen, CA, USA) with NIR-780 excitation and Emission filters at an exposure value of 10 seconds.

***b) Ex vivo Imaging***: The animals were sacrificed one week after i.v. injection and major organs such as heart, liver, lungs, spleen, and kidneys, and intestine were collected along with the tumor samples for imaging using IVIS machine (xenogen).

***c) In vivo Therapeutic Efficacy***: The preclinical assessment in animals using Balb/c mice was done at KAIST as per the KAIST animal ethical committee rules and regulations. Female BALB/c nude mice (5-week-old) were injected with 5 x 10^5^ cells/200 μL of 4T1 cells subcutaneously into the right hind flank. Next, IR 780 (Sigma, USA) with excitation and emission wavelengths of 778 and NIR-780 doped NPs were diluted in 0.9% saline and administered via tail vein injection. The mice were randomly separated into six groups including a control group (non-injection). Each group was intravenously injected with 200 μl of free PTX (10 mg/kg), PBS, PTX:Mn:ZnS NPs; PTX:PEN/Mn:ZnS NPs; PTX:pVEC/Mn:ZnS NPs; PTX:R9/Mn:ZnS NPs once per week (~7.5mg/kg, where PTX concentration was retained as ~ 0.3 mg/kg). The body weights of the groups were measured, and the tumor volumes were calculated as follows: V = WL^2^ /2 (W= the longest diameter; L= the shortest diameter). In addition, mouse organs (liver, kidneys, heart, spleen, and lungs) with tumors were collected and stained with hematoxylin and eosin (H&E) to test for toxicity using an optical microscope.

***File S7. Analytical Determinations***

The particle size and size distribution were measured with a dynamic light scattering (DLS) method (Zetasizer nano, Malvern, UK) carried out with a He–Ne (633 nm) and 90 collecting optics at room temperature (RT). The samples were prepared with 1 mg/mL concentration. TEM was conducted using a 120 kV FEI Technai G2 Bio-TEM. To prepare the TEM sample, 0.1 mg/mL of the nanoparticle solution was dropped onto a 230 mesh copper grid with carbon, and then negatively stained with 2% uranyl acetate solution. The scanning electron microscopic images were obtained at the KAIST, KARA facility with 0.1mg/mL concentration in distilled water. The copper grid was dried by a heater within a minute. The fluorescence spectral studies were done using a Pl-F350/PC spectro fluoro-photometer-Shimadzu using 0.1mg/mL concentrations. *In vivo* live imaging followed by *ex vivo* tissue imaging was done using IVIS Lumina (Xenogen, CA) with ICG excitation and emission filters at an exposure value of 10 seconds.


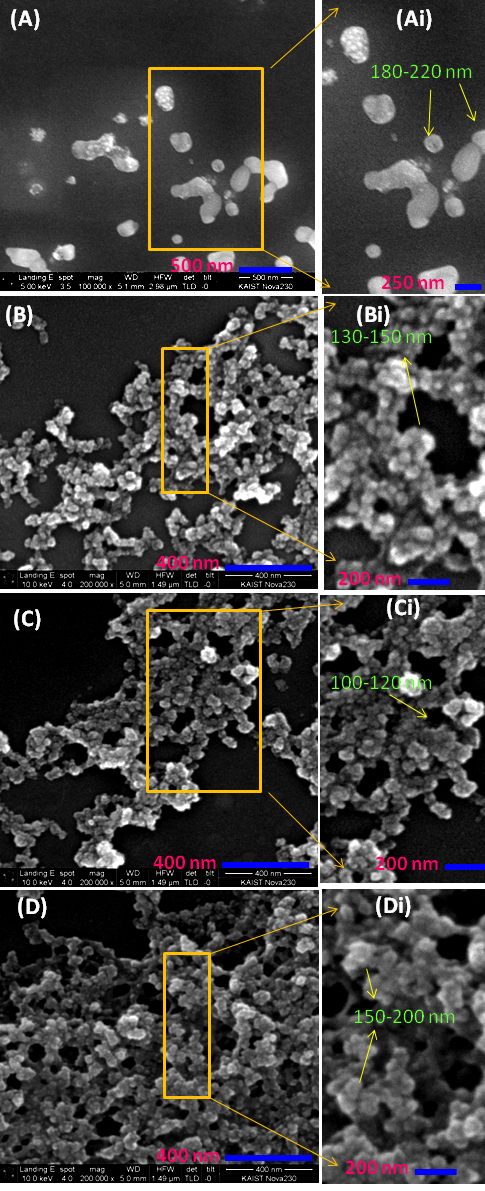

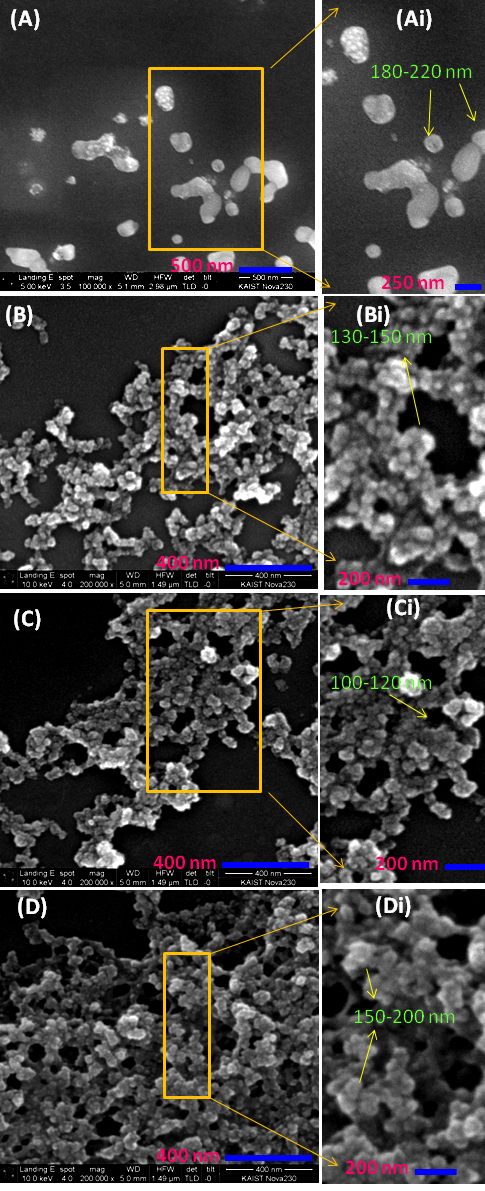


**Figure S1.** SEM analysis of different PTX loaded NPs: (A) PTX/Mn:ZnS NPs; (B) PTX-PEN/Mn: ZnS NPs; (C) PTX-pVEC:Mn: ZnS NPs; and (D) PTX/R9: Mn:ZnS NPs; Ai, Bi, Ci and Di represents their respective magnified version in SEM.

***
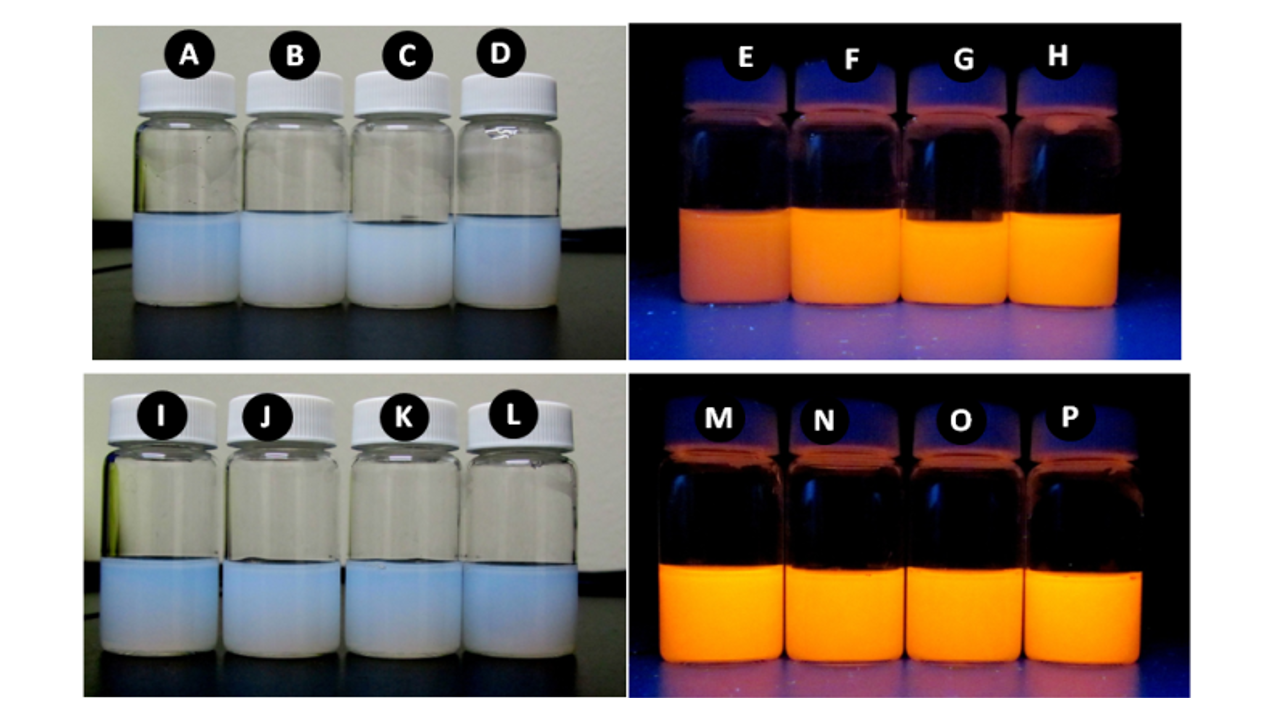
***

**Figure S2.** Optical micrographs for (A) the stable Mn: ZnS NPs; (B) PEN/Mn:ZnS NPs; (C) pVEC/Mn: ZnS NPs; (D) R9/Mn:ZnS NPs under normal light; (E)-(H) under UV light; (I)-(L) representative images for the PTX:CPP modified NPs under normal light and (M) –(P) respective images under UV light.

***
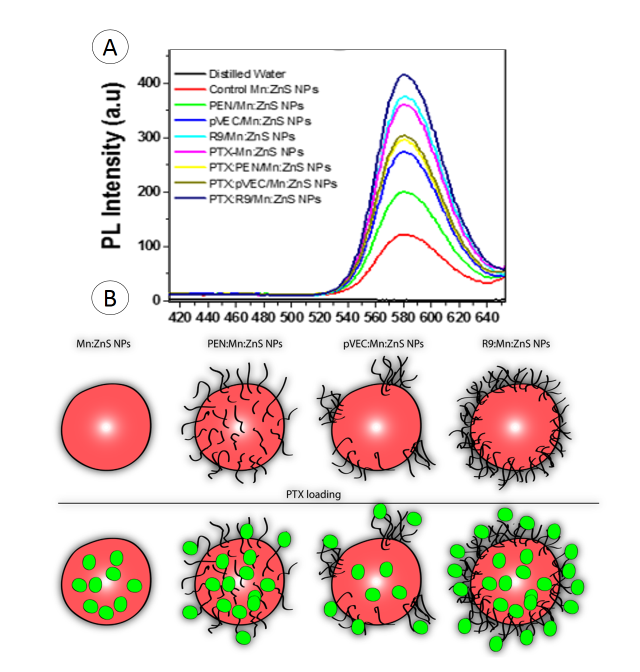
***

**Figure S3.** Photoluminescence spectra for the PTX loaded and bare CPP modified Mn: ZnS NPs; (H) PTX interaction with Mn:ZnS NPs and their CPP modified versions.


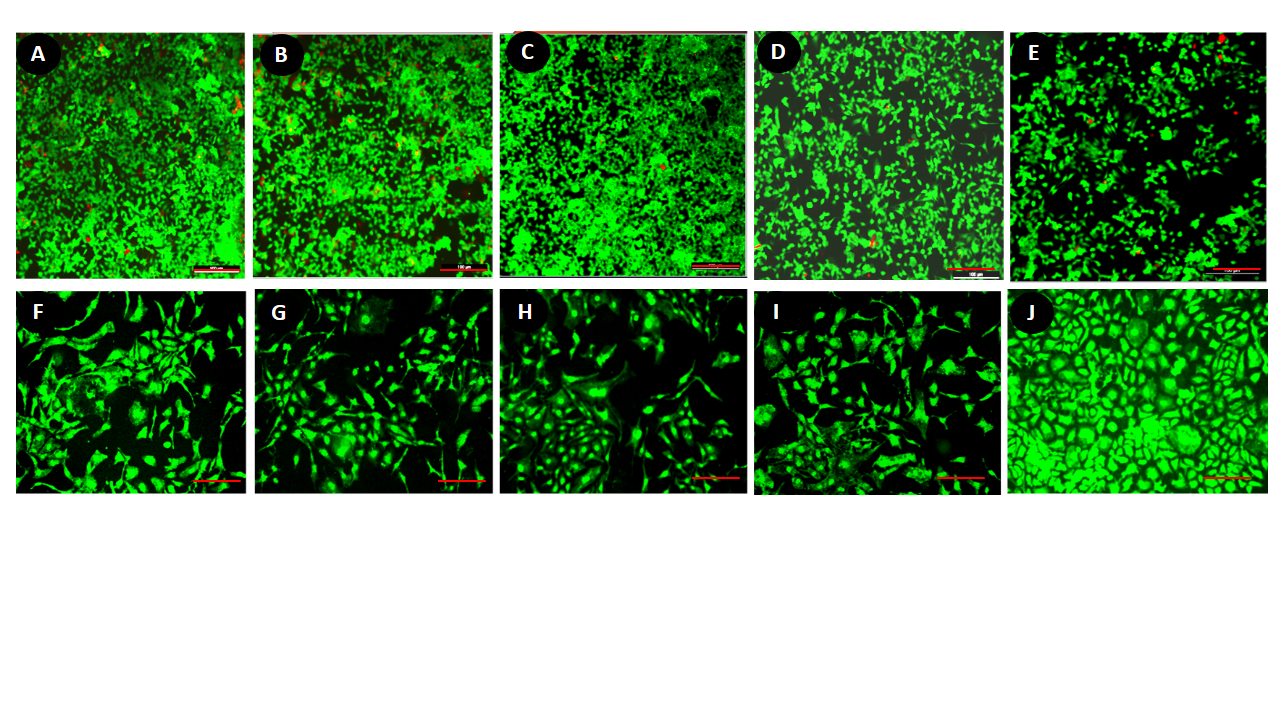


**Figure S4**. Live/dead assay on CPP modified NPs after 48h incubation period: (A) Control HeLa cells; (B)Mn:ZnS NPs; (C) PEN/Mn:ZnS NPs; (D) pVEC/Mn: ZnS NPs; (E) R9/Mn:ZnS NPs ; (F) Control SKOV-3 cells; (G) Mn: ZnS NPs; (H) PEN/Mn: ZnS NPs; (I) pVEC/Mn: ZnS NPs; (J) R9/Mn: ZnS NPs respectively (scale bar represents 100 µm)


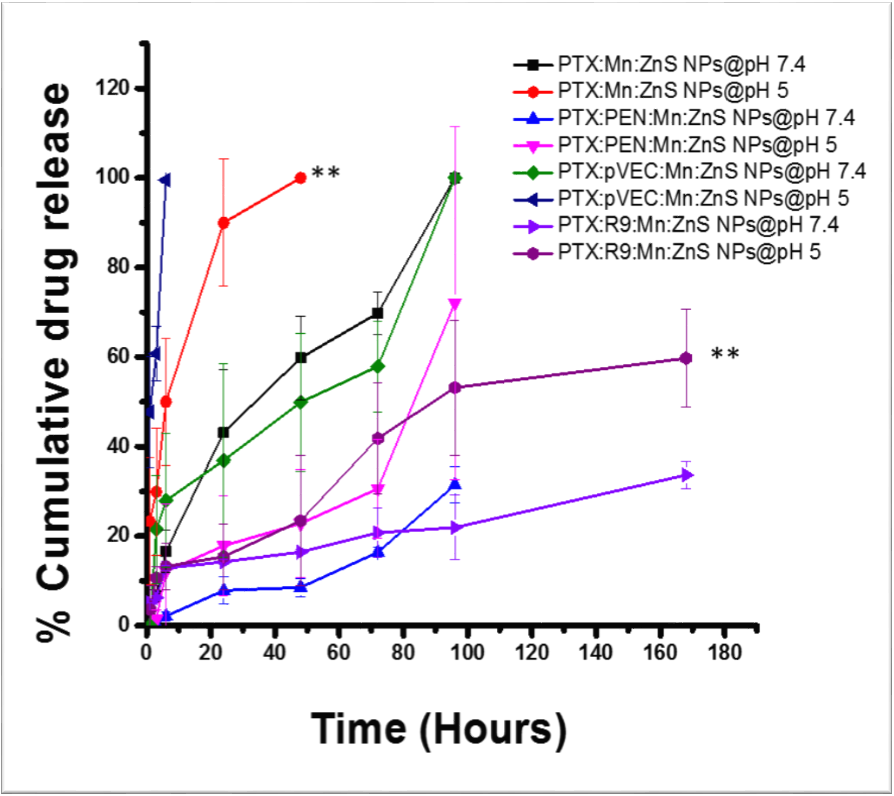


**Figure S5**. PTX release at pH 5 and 7.4 from Mn: ZnS NPs, PEN: Mn: ZnS NPS; pVEC/ Mn: ZnS NPs; and R9/Mn:ZnS NPs respectively (n=3, ** p<0.01)

**
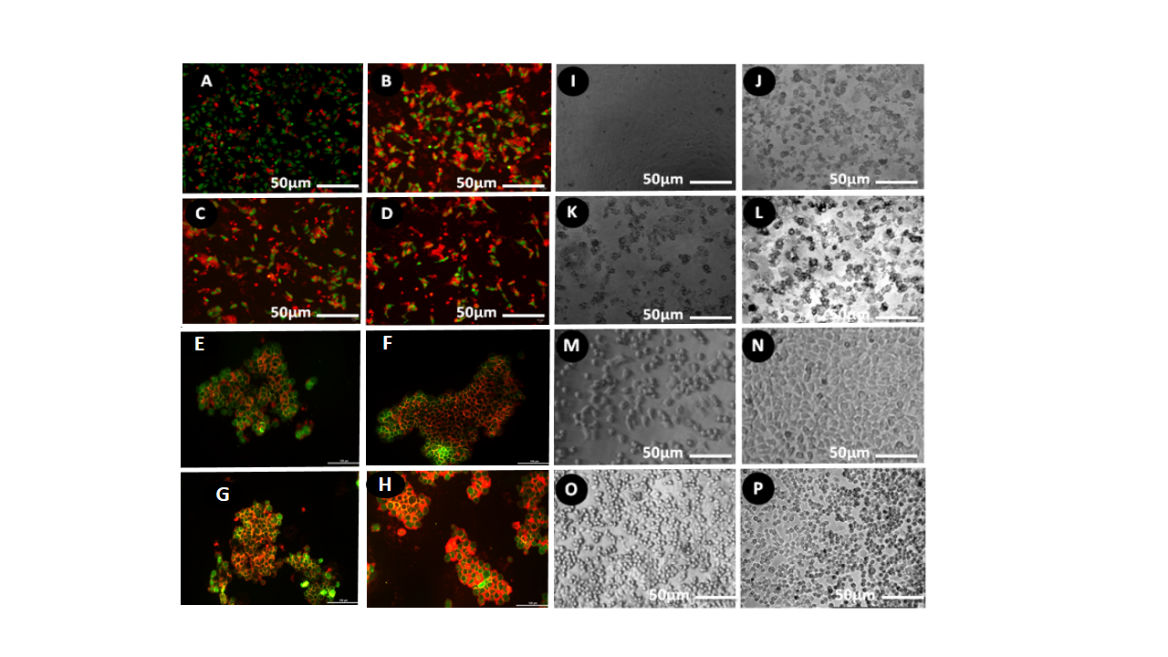
**

**Figure S6.** Live/dead assay: anti-cancer efficacy of CPP modified and unmodified NPs on SKOV3 and HeLa cells: (A) Mn:ZnS NPs; (B) PEN/Mn:ZnS NPs; (C) pVEC/Mn:ZnS NPs; (D) R9/Mn:ZnS NPs in SKOV3 cells; (E)-(H) on HeLa cells; (I)-(L) are the optical micrographs for the CPP modified NPs on SKOV3 and HeLa cells (M-P) respectively.


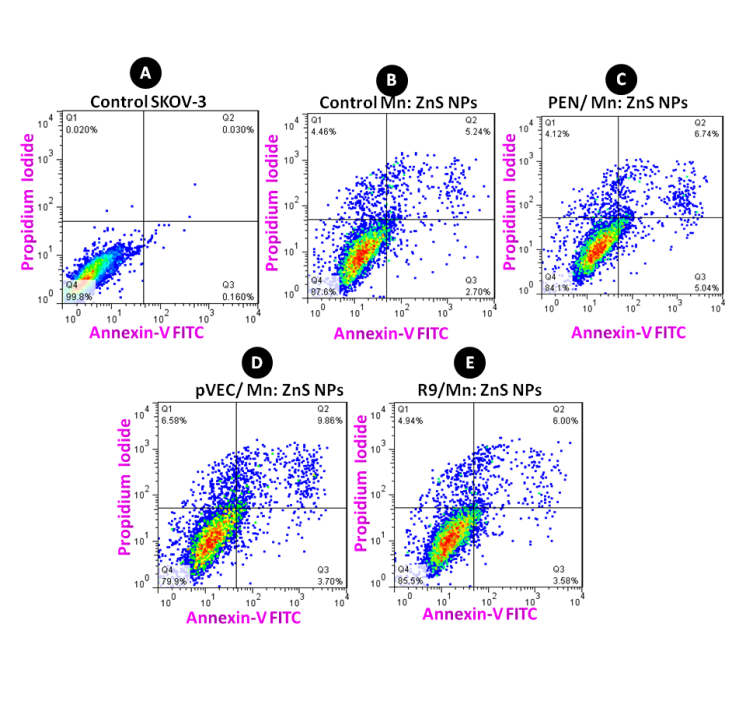


**Figure S7.** The non-apoptotic profile on SKOV-3 cells by (A) Mn: ZnS NPs; (B) PEN/ Mn: ZnS NPs; (C) pVEC/Mn: ZnS NPs; (D) R9/Mn: ZnS NPs after 48h incubation time.


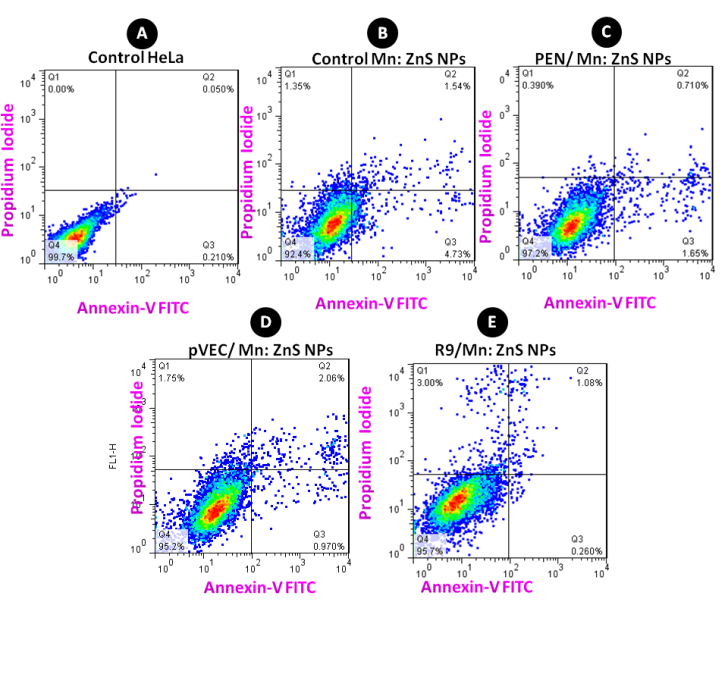


**Figure S8.**The non-apoptotic profile on HeLa cells by (A) Mn:ZnS NPs; (B) PEN/ Mn:ZnS NPs; (C) pVEC/Mn:ZnS NPs; (D) R9/Mn:ZnS NPs after 48h incubation time.


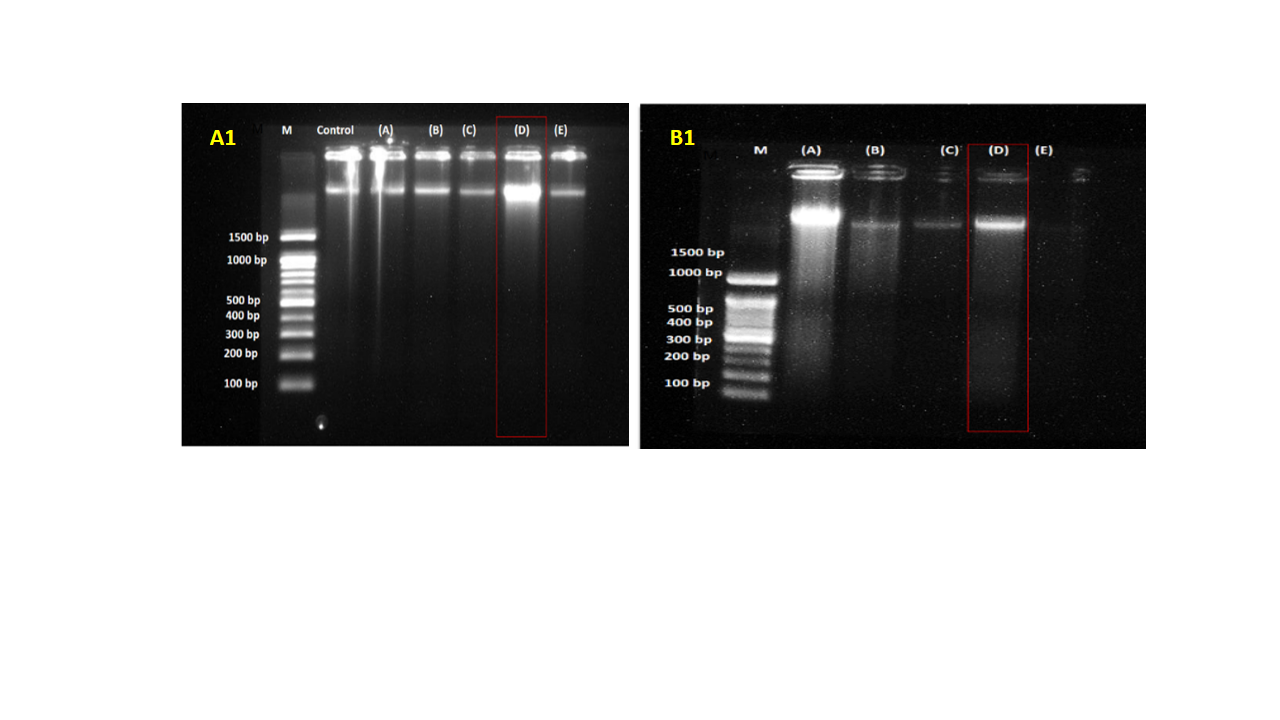


**Figure S9A1.** The DNA fragmentation assay on HeLa cells after 48 h incubation of PTX loaded CPP modified NPs. (M) 100 bp marker; (A) PTX-Mn: ZnS NPs; (B) PEN/PTX-Mn: ZnS NPs; (C) pVEC/PTX-Mn: ZnS NPs; (D) R9/PTX-Mn: ZnS NPs;(E) bare Mn: ZnS NPs after 48h incubation time. B1: The DNA fragmentation assay on SKOV-3 cells after 48 h incubation of PTX loaded CPP modified NPs. (M) 100 bp marker; (A) PTX alone; (B) PEN/PTX-Mn: ZnS NPs; (C) pVEC/PTX-Mn: ZnS NPs; (D) R9/PTX-Mn: ZnS NPs;(E) bare Mn: ZnS NPs after 48h incubation time.


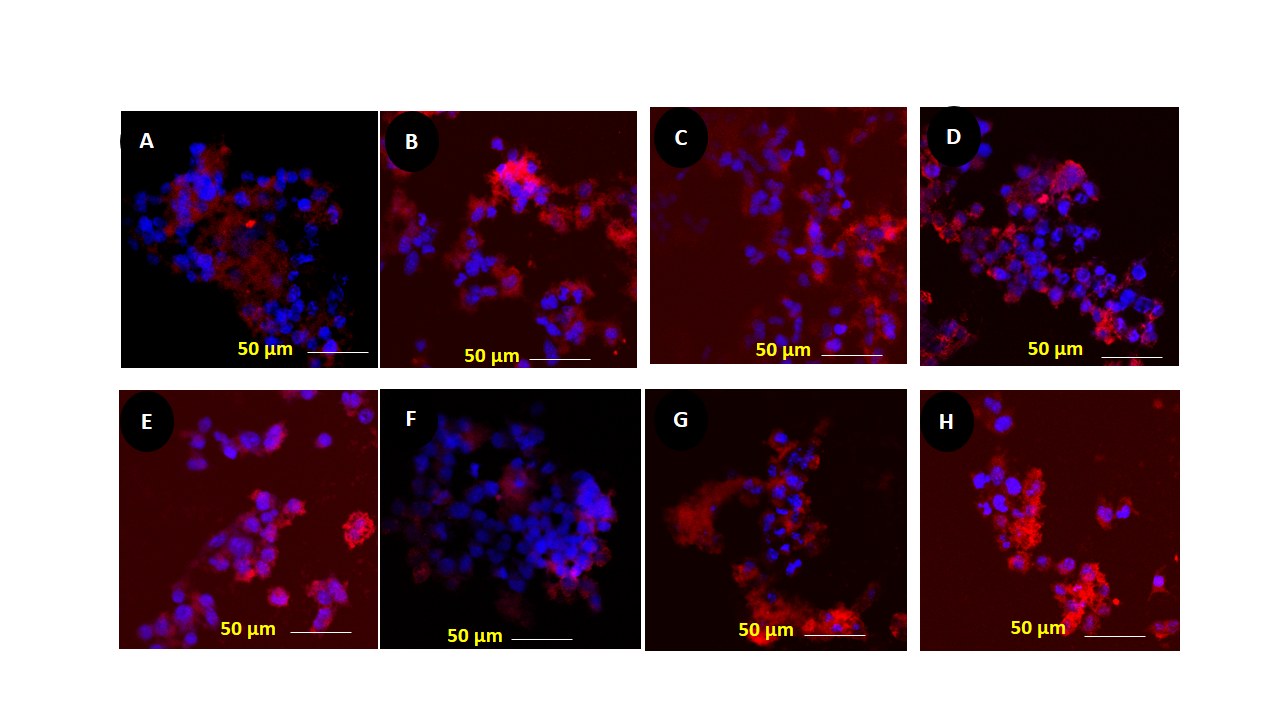


**Figure S10.** (A-D) represents the CT26 cellular uptake of PTX: Mn:ZnS NPs; PTX/PEN:Mn: ZnS NPs; PTX/pVEC: Mn:ZnS NPs and PTX/R9:Mn:ZnS NPs; (E-H) their corresponding PTX loaded NPs after 48 h incubation: The blue colour indicates the nuclear staining using API and red fluorescence is from Mn:ZnS NPs respectively.


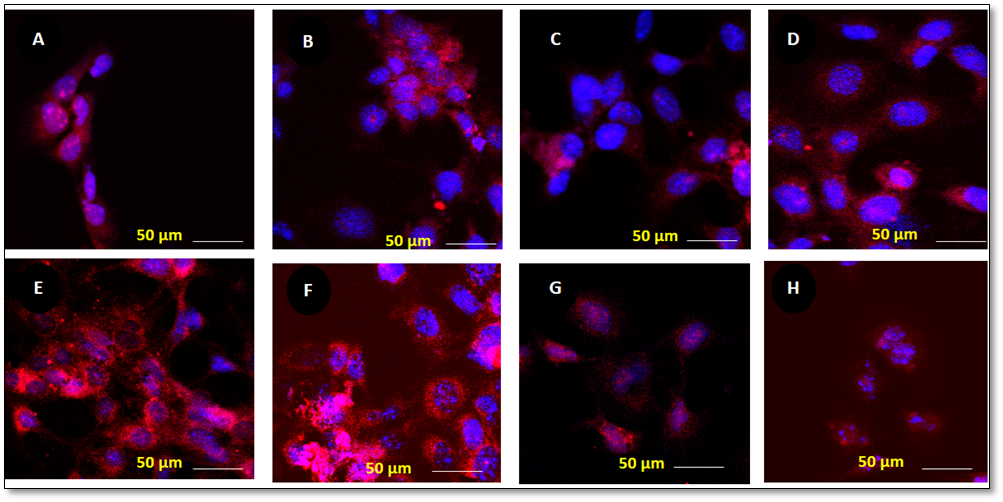


**Figure S11.** (A-D) represents the 4T1 cellular uptake of PTX: Mn: ZnS NPs; PTX/PEN: Mn:ZnS NPs; PTX/pVEC:Mn: ZnS NPs and PTX/R9:Mn:ZnS NPs; (E-H) their corresponding PTX loaded NPs after 48h incubation: The blue colour indicates the nuclear staining using API and red fluorescence is from Mn:ZnS NPs respectively.


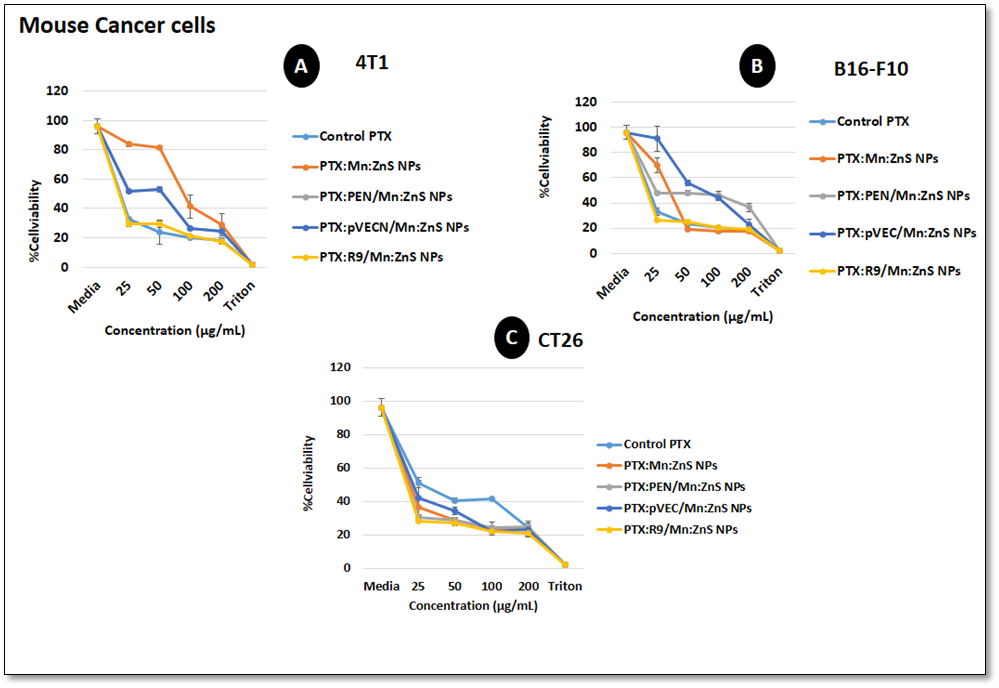


**Figure S12.** Anti-cancer efficacy of PTX: Mn: ZnS NPs; PTX:PEN/Mn: ZnS NPs; PTX: pVEC/ Mn: ZnS NPs and PTX: R9/Mn:ZnS NPs on (A) 4T1; (B) B16-F10 and (C) CT26 cell lines after 48 h of incubation period.


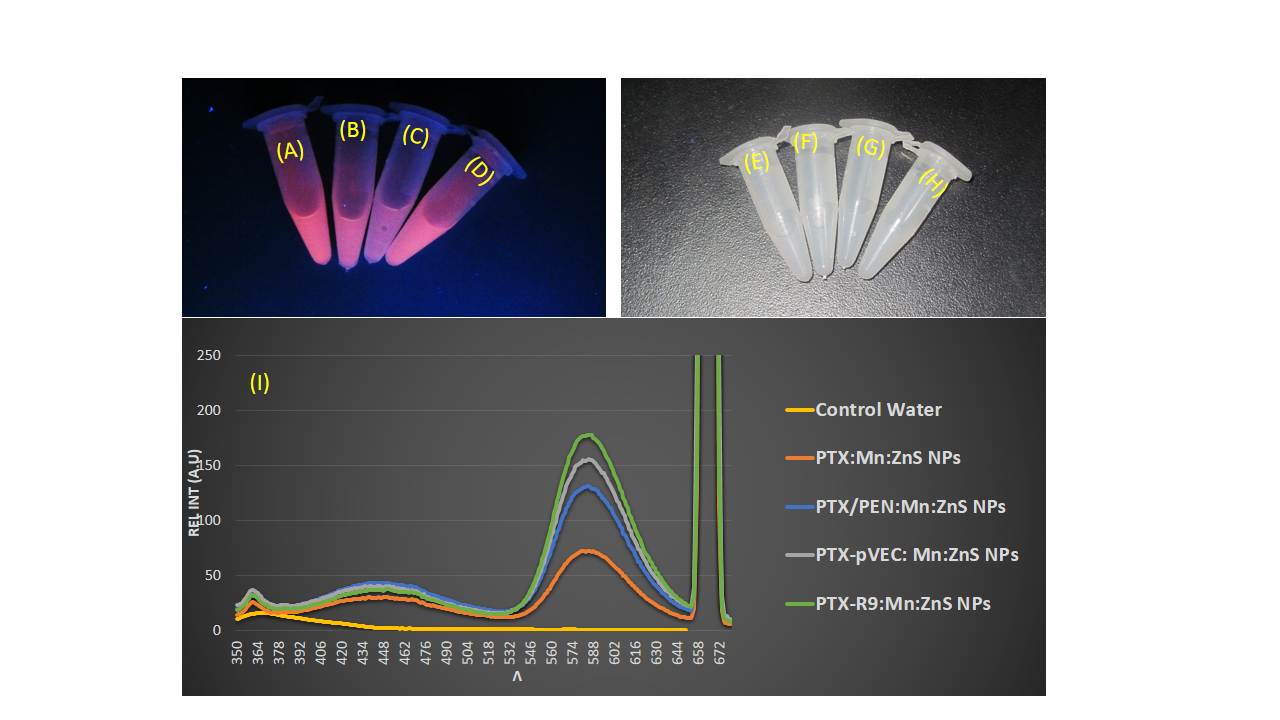


**Figure S13.** (A-D) represents the UV exposed PTX: Mn: ZnS NPs; PTX:PEN/Mn: ZnS NPs; PTX: pVEC/Mn:ZnS NPs and PTX: R9/Mn: ZnS NPs; (E-H) their corresponding bright light images respectively 30 days after incubation with 10% FBS contained PBS at 37°C. (I) represents their PL spectra indicating there is no quenching in the fluorescence of Mn:ZnS NPs; PEN/Mn:ZnS NPs; pVEC/Mn: ZnS NPs and R9/Mn: ZnS NPs after 30-day incubation time.


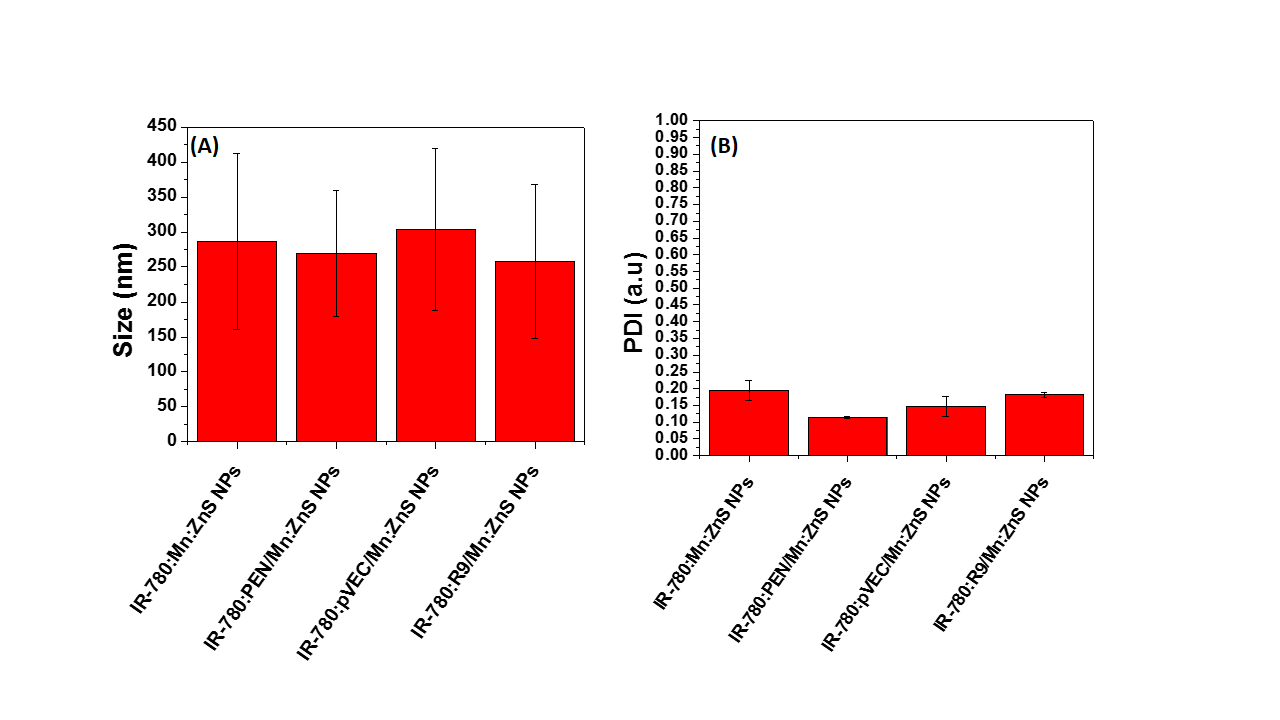


**Figure S14.** Size analysis of IR-780 loaded Mn:ZnS NPs and its CPP modified versions: (A) DLS and (B) PDI indices for IR-780:Mn:ZnS NPs; IR-780:PEN:Mn:ZnS NPs; IR-780:pVEC:Mn:ZnS NPs; IR-780:R9:Mn:ZnS NPs respectively.


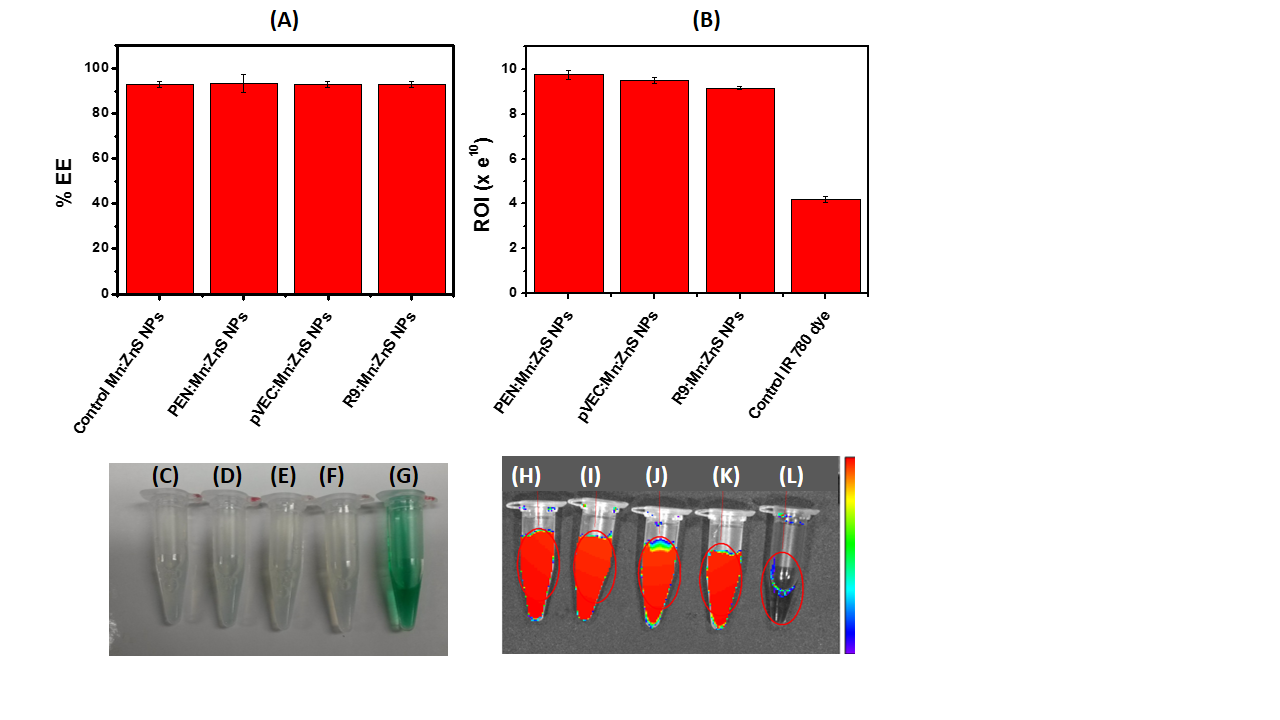


**Figure S15.** (A) NIR-780 dye encapsulation efficiency in doped samples; (B) *In vitro* NIR fluorescence analysis (region of interest= ROI); (C-G) optical photographs of Mn: ZnS NPs; PEN/ Mn: ZnS NPs; pVEC/Mn: ZnS NPs, R9/Mn: ZnS NPs and control IR-780 dye and (H-J) represents their NIR images indicating there is no quenching in the fluorescence.

**Table S1. Loading efficiency and drug contents in CPP modified Mn:ZNs NPs**

| Samples | Loading Efficiency (LE) | Drug Content (µM) |
| --- | --- | --- |
| PTX:Mn:ZnS NPs | 2.4±0.3 | 2.88 |
| PTX:PEN-Mn:ZnS NPs | 1.8.3±0.4 | 2.16 |
| PTX:pVEC/Mn:ZnS NPs | 2.1±0.3 | 2.52 |
| PTX:R9/Mn:ZnS NPs | 3.0±0.7 | 3.6 |

**References:**

N. S. Rejinold, T. Baby, K. P. Chennazhi and R. Jayakumar, **Dual drug encapsulated thermo-sensitive fibrinogen-graft-poly (N-isopropyl acrylamide) nanogels for breast cancer therapy**. *Colloids Surf B Biointerfaces*. 2014;114:209-17

N. S. Rejinold, T. Baby, S. V. Nair and R. Jayakumar, **Paclitaxel loaded fibrinogen coated CdTe/ZnTe core shell nanoparticles for targeted imaging and drug delivery to breast cancer cells**. *J Biomed Nanotechnol*. 2013;9:1657-71

N. S. Rejinold, R. G. Thomas, M. Muthiah, K. P. Chennazhi, I. K. Park, Y. Y. Jeong, et al., **Radio frequency triggered curcumin delivery from thermo and pH responsive nanoparticles containing gold nanoparticles and its in vivo localization studies in an orthotopic breast tumor model**. *Rsc Adv*. 2014;4:39408-39427
